# Supplementary material for: Paired Electrolysis Enabled Cyanation of Diaryl Diselenides with KSCN Leading to Aryl Selenocyanates
Source: Molecules. 2023 Feb 1;28(3):1397. doi: 10.3390/molecules28031397 (PMC9919590; doi:10.3390/molecules28031397)
Supplement: Supplementary file 1 [file molecules-28-01397-s001.zip › molecules-2151722-supplementary.pdf]

Supporting Information

Paired Electrolysis Enabled Cyanation of Diaryl Diselenides with KSCN Leading to Aryl Selenocyanates

Wei-Bao He [1], Luo-Lin Tang [2], Jun Jiang [2], Xiao Li [2], Xinhua Xu [1,*] Tian-Bao Yang [3], Wei-Min He [2,*]

1 College of Chemistry and Chemical Engineering, Hunan University, Changsha 410082, China

2 School of Chemistry and Chemical Engineering, University of South China, Hengyang 421001, China

3 National Engineering Research Center of Low-Carbon Processing and Utilization of Forest Biomass, Nanjing Forestry University, Nanjing 210037, China

* Correspondence: xhx1581@hnu.edu.cn (X.X.); weiminhe@usc.edu.cn (W.-M.H.)

Table of Content

1. General Information……………………………………………………..S2

2. Experimental Section……………………………………………………….S3

3. Characterization Data of Products……………………………………...S4

4. References…………………………………………………………………….S8

5. ^{1}H and ^{13}C NMR Spectra of Products……………………………..S9

1. General Information

Unless otherwise noted, all reagents were obtained from commercial suppliers and used without further purification. The instrument for electrolysis is dual display potentiostat (DJS-292B) (made in China). Graphite electrode is 15 mm × 10 mm × 2 mm and platinum electrode is 15 mm × 10 mm × 0.1 mm. The instrument for cyclic voltammetrys is CHI 660E potentiostat, and the conditions are as follow: a glassy carbon disk working electrode (diameter, 2 mm), Pt disk and Ag/AgCl (0.1 M in CH_3CN) as counter and reference electrode. Thin layer chromatography (TLC) employed glass 0.25 mm silica gel plates. Flash chromatography columns were packed with 200-300 mesh silica gel. ^1H NMR spectra were recorded at 500 MHz and ^{13}C NMR spectra were recorded at 126 MHz by using a Bruker Avance 500 spectrometer. Chemical shifts were calibrated using residual undeuterated solvent as an internal reference (^1H NMR: $CDCl_3$ 7.26 ppm, ^{13}C NMR: $CDCl_3$ 77.0 ppm), the chemical shifts (δ) were expressed in ppm and J values were given in Hz. HRMS were performed on a spectrometer operating on ESI-TOF.

2. Experimental Section

2.1 General experimental procedures for Compound 3

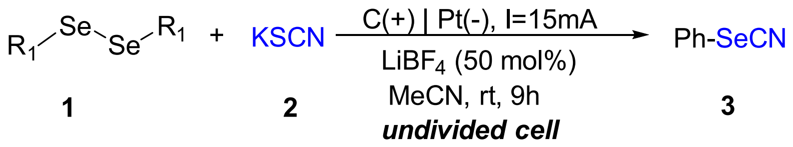

In an undivided flask (10 mL) equipped with a stir bar, diphenyl diselenide (0.2 mmol), KSCN (0.5 mmol), $LiBF_4$ (0.1 mmol) and MeCN (6 mL) were added. The flask was equipped with graphite (15 mm × 10 mm × 2 mm) as the anode and platinum electrode (15 mm × 10 mm × 0.1 mm) as the cathode. The reaction mixture was stirred and electrolyzed at a constant current of 15 mA under room temperature for 9 h. After completion, the solvent was concentrated under reduced pressure, and the pure products **3** were obtained by flash chromatography on silica gel.

2.2 Cyclic voltammetry experiment:

CV measurements were performed on a CHI 660E potentiostat, and the conditions are as follow: a glassy carbon disk working electrode (diameter, 2 mm), Pt disk and Ag/AgCl (0.1 M in MeCN) as counter and reference electrode. Cyclic voltammograms of reactants and their mixtures in 0.1 M $LiBF_4$/MeCN using a glassy carbon disk working electrode (diameter, 2 mm), Pt disk and Ag/AgCl (0.1 M in MeCN) as counter and reference electrode at 50 mV/s scan rate: a) **1a** (20 mmol/L) (orange line), b) **2a** (20 mmol/L) (purple line), c) **1a** (20 mmol/L) and **2a** (20 mmol/L), (red line).

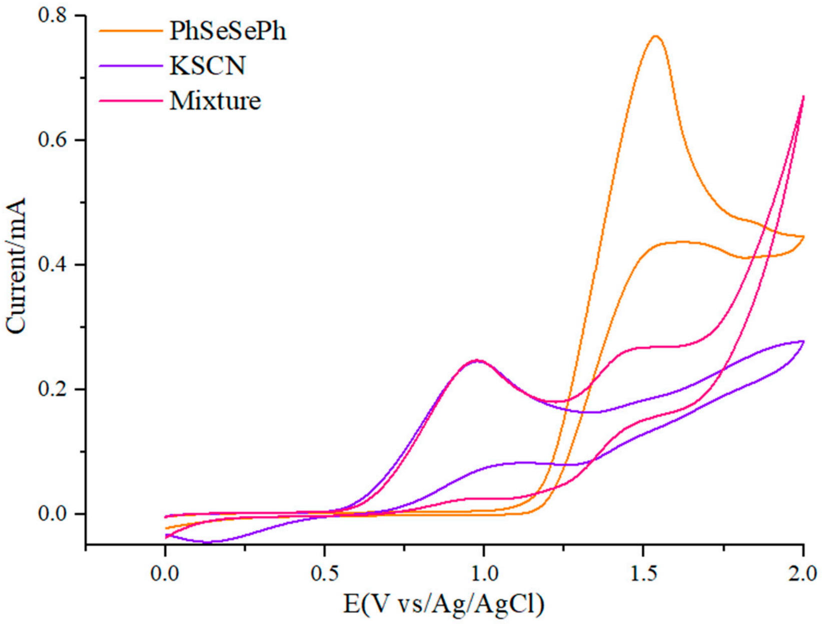

Figure S1 Cyclic voltammetry experiment

3. Characterization data of products 3a-3x

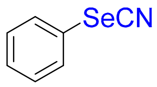

*Selenocyanatobenzene (**3a**)*[19]: Prepared following general procedure and the reaction mixture was purified by flash column chromatography to afford the product **3a** (29.7 mg, 81% yield). Colorless liquid. ^{1}H NMR (500 MHz, $CDCl_3$) δ 7.57 (d, J = 7.2 Hz, 2H), 7.34 (m, 3H).^{13}C NMR (126 MHz, $CDCl_3$) δ 131.76, 129.37, 128.76, 120.79, 100.48.

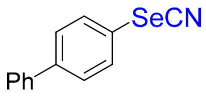

*4-selenocyanato-1,1′-biphenyl (**3b**)*[16]: Prepared following general procedure and the reaction mixture was purified by flash column chromatography to afford the product **3b**(33.7 mg, 65% yield). White solid.^{1}H NMR (500 MHz, $CDCl_3$) δ 7.60 (d, J = 8.1 Hz, 2H), 7.51 (d, J = 8.2 Hz, 2H), 7.47 (d, J = 7.7 Hz, 2H), 7.37 (t, J = 7.5 Hz, 2H), 7.31 (d, J = 7.3 Hz, 1H). ^{13}C NMR (126 MHz, $CDCl_3$) δ 141.96, 138.40, 132.24, 127.98, 127.15, 126.09, 119.38, 100.34.

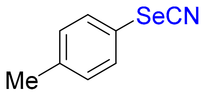

*1-methyl-4-selenocyanatobenzene (**3c**)*[19]: Prepared following general procedure and the reaction mixture was purified by flash column chromatography to afford the product **3c**(28.8 mg, 73% yield). Yellow liquid. ^{1}H NMR (500 MHz, $CDCl_3$) δ 7.46 (d, J = 7.8 Hz, 2H), 7.14 (d, J = 7.9 Hz, 2H), 2.31 (s, 3H).^{13}C NMR (126 MHz, $CDCl_3$) δ 139.42, 132.31, 130.12, 116.87, 100.80, 20.20.

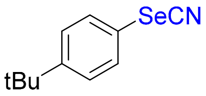

*1-(tert-butyl)-4-selenocyanatobenzene (**3d**)*[15]: Prepared following general procedure and the reaction mixture was purified by flash column chromatography to afford the product **3d**(34.4 mg, 72% yield). Colorless liquid. ^{1}H NMR (500 MHz, $CDCl_3$) δ 7.49 (d, J = 8.4 Hz, 2H), 7.34 (d, J = 8.4 Hz, 2H), 1.24 (s, 9H).^{13}C NMR (126 MHz, $CDCl_3$) δ 152.51, 132.02, 126.52, 116.99, 100.77, 33.79, 30.09.

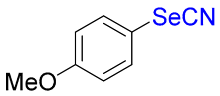

*1-methoxy-4-selenocyanatobenzene (**3e**)*[19]: Prepared following general procedure and the reaction mixture was purified by flash column chromatography to afford the product **3e** (33.2mg, 78% yield). Colorless liquid.^{1}H NMR (500 MHz, $CDCl_3$) δ 7.50 (d, J = 8.7 Hz, 2H), 6.82 (d, J = 8.8 Hz, 2H), 3.72 (s, 3H). ^{13}C NMR (126 MHz, $CDCl_3$) δ 160.30, 134.96, 114.98, 110.03, 101.10, 54.46.

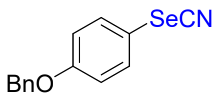

*1-(benzyloxy)-4-selenocyanatobenzene (**3f**)*: Prepared following general procedure and the reaction mixture was purified by flash column chromatography to afford the product **3f** (43.9mg, 76% yield).Yellow solid. ^{1}H NMR (500 MHz, $CDCl_3$) δ 7.47 (d, J = 8.7 Hz, 2H), 7.30 (d, J = 6.9 Hz, 4H), 7.24 (t, J = 6.5 Hz, 1H), 6.87 (d, J = 8.8 Hz, 2H), 4.96 (s, 2H).^{13}C NMR (126 MHz, $CDCl_3$) δ 159.36, 135.00, 134.94, 127.66, 127.23, 126.40, 115.81, 110.30, 101.08, 69.11. HRMS: calculated for $C_{14}H_{11}NOSeNa$ $[M+Na]^+$: 311.9898, found: 311.9900.

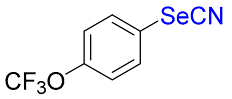

*1-selenocyanato-4-(trifluoromethoxy)benzene (**3g**)*[15]: Prepared following general procedure and the reaction mixture was purified by flash column chromatography to afford the product **3g** (38.4mg, 72% yield).Yellow solid. ^{1}H NMR (500 MHz, $CDCl_3$) δ 7.60 (d, J = 8.4 Hz, 2H), 7.17 (d, J = 8.5 Hz, 2H). ^{13}C NMR (126 MHz, $CDCl_3$) δ 149.40, 133.66, 121.70, 119.27(q, J = 259.2 Hz), 118.73, 100.03.

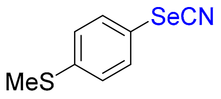

*methyl(4-selenocyanatophenyl)sulfane (**3h**)* [15]: Prepared following general procedure and the reaction mixture was purified by flash column chromatography to afford the product **3h**(32.1 mg, 70% yield).Yellow solid. ^{1}H NMR (500 MHz, $CDCl_3$) δ 7.37 (d, J = 8.5 Hz, 2H), 7.05 (d, J = 8.5 Hz, 2H), 2.32 (s, 3H).^{13}C NMR (126 MHz, $CDCl_3$) δ 142.26, 133.82, 127.26, 116.75, 102.04, 15.17.

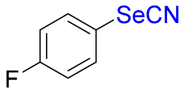

*1-fluoro-4-selenocyanatobenzene (**3i**)*[15]: Prepared following general procedure and the reaction mixture was purified by flash column chromatography to afford the product **3i**(26.5mg, 66% yield). Colorless liquid. ^{1}H NMR (500 MHz, $CDCl_3$) δ 7.58 (dd, J = 8.9, 5.0 Hz, 2H), 7.03 (t, J = 8.6 Hz, 2H). ^{13}C NMR (126 MHz, $CDCl_3$) δ 162.77(d, J = 252.2 Hz), 134.79 (d, J = 8.5 Hz), 116.75 (d, J = 22.4 Hz), 115.08 (d, J = 3.5 Hz), 100.39.

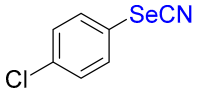

*1-chloro-4-selenocyanatobenzene (**3j**)*[15]: Prepared following general procedure and the reaction mixture was purified by flash column chromatography to afford the product **3j**(26.5mg, 61% yield). Yellow solid. ^{1}H NMR (500 MHz, $CDCl_3$) δ 7.50 (d, J = 8.7 Hz, 2H), 7.31 (d, J = 8.5 Hz, 2H).^{13}C NMR (126 MHz, $CDCl_3$) δ 135.47, 133.19, 129.57, 118.71, 100.03.

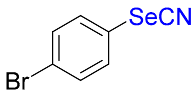

*1-bromo-4-selenocyanatobenzene (**3k**)*[15]: Prepared following general procedure and the reaction mixture was purified by flash column chromatography to afford the product **3k**(35.0 mg, 67% yield). White solid. ^{1}H NMR (500 MHz, $CDCl_3$) δ 7.45 (d, J = 8.5 Hz, 2H), 7.42 (d, J = 8.6 Hz, 2H). ^{13}C NMR (126 MHz, $CDCl_3$) δ 133.25, 132.49, 123.53, 119.48, 99.90.

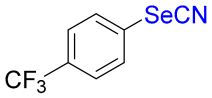

*1-selenocyanato-4-(trifluoromethyl)benzene (**3l**)*[15]: Prepared following general procedure and the reaction mixture was purified by flash column chromatography to afford the product **3l**(34.1 mg, 68% yield). Colorless liquid. ^{1}H NMR (500 MHz, $CDCl_3$) δ 7.45 (d, J = 8.5 Hz, 2H), 7.42 (d, J = 8.6 Hz, 2H). ^{13}C NMR (126 MHz, $CDCl_3$) δ 130.96, 130.67 (q, J = 33.2 Hz), 126.16 (q, J = 3.7 Hz), 125.91, 124.60 (q, J =273.0 Hz), 99.45.

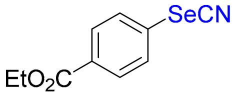

*ethyl 4-selenocyanatobenzoate (**3m**)*[15]: Prepared following general procedure and the reaction mixture was purified by flash column chromatography to afford the product **3m**(35.2 mg, 69% yield). White solid. ^{1}H NMR (500 MHz, $CDCl_3$) δ 7.98 (d, J = 8.3 Hz, 2H), 7.60 (d, J = 8.3 Hz, 2H), 4.32 (q, J = 7.1 Hz, 2H), 1.33 (t, J = 7.1 Hz, 3H).^{13}C NMR (126 MHz, $CDCl_3$) δ 164.39, 130.42, 130.21, 130.19, 126.87, 99.50, 60.50, 13.24.

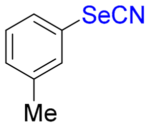

*1-methyl-3-selenocyanatobenzene (**3n**)*[16]: Prepared following general procedure and the reaction mixture was purified by flash column chromatography to afford the product **3n**(28.8mg, 73% yield). Yellow liquid. ^{1}H NMR (500 MHz, $CDCl_3$) δ 7.37 (s, 1H), 7.34 (d, J = 7.8 Hz, 1H), 7.20 (t, J = 7.7 Hz, 1H), 7.14 (d, J = 7.7 Hz, 1H), 2.29 (s, 3H).^{13}C NMR (126 MHz, $CDCl_3$) δ 139.59, 132.20, 129.56, 129.07, 128.66, 120.45, 100.70, 20.26.

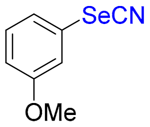

*methoxy-3-selenocyanatobenzene (**3o**)*[16]: Prepared following general procedure and the reaction mixture was purified by flash column chromatography to afford the product **3o**(29.8mg, 70% yield). Yellow liquid. ^{1}H NMR (500 MHz, $CDCl_3$) δ 7.29 (t, J = 8.0 Hz, 1H), 7.20 – 7.12 (m,

2H), 6.96 – 6.91 (m, 1H), 3.81 (s, 3H). ^{13}C NMR (126 MHz, $CDCl_3$) δ 160.64, 131.07, 124.58, 122.50, 117.86, 115.76, 101.35, 55.56.

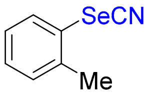

*1-methyl-2-selenocyanatobenzene (**3p**)*[19]: Prepared following general procedure and the reaction mixture was purified by flash column chromatography to afford the product **3p**(25.2mg, 64% yield). Colorless liquid. ^{1}H NMR (500 MHz, $CDCl_3$) δ 7.63 (d, J = 7.8 Hz, 1H), 7.29 – 7.21 (m, 2H), 7.14 (t, J = 7.5 Hz, 1H), 2.41 (s, 3H). ^{13}C NMR (126 MHz, $CDCl_3$) δ 138.86, 132.95, 130.18, 129.30, 126.83, 121.97, 100.19, 21.28.

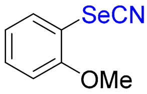

*1-methoxy-2-selenocyanatobenzene (**3q**)*[19]: Prepared following general procedure and the reaction mixture was purified by flash column chromatography to afford the product **3q**(26.4mg, 62% yield). Colorless liquid. ^{1}H NMR (500 MHz, $CDCl_3$) δ 7.53 (d, J = 7.8 Hz, 1H), 7.25 (t, J = 7.8 Hz, 1H), 6.93 (t, J = 7.6 Hz, 1H), 6.80 (d, J = 8.3 Hz, 1H), 3.79 (s, 3H). ^{13}C NMR (126 MHz, $CDCl_3$) δ 154.74, 129.01,128.88, 121.80, 111.59, 110.04, 100.39, 55.21.

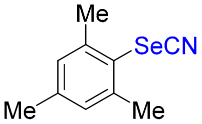

*1,3,5-trimethyl-2-selenocyanatobenzene (**3r**)*[15]: Prepared following general procedure and the reaction mixture was purified by flash column chromatography to afford the product **3r**(33.3 mg, 74% yield). White solid. ^{1}H NMR (500 MHz, $CDCl_3$) δ 6.85 (s, 2H), 2.41 (s, 6H), 2.15 (s, 3H). ^{13}C NMR (126 MHz, $CDCl_3$) δ 142.86, 141.28, 129.71, 120.52, 101.45, 24.38, 21.08.

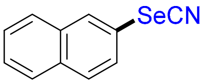

*2-selenocyanatonaphthalene (**3s**)*[19]: Prepared following general procedure and the reaction mixture was purified by flash column chromatography to afford the product **3s**(30.8 mg, 66% yield). White solid. ^{1}H NMR (500 MHz, $CDCl_3$) δ 7.82 (s, 1H), 7.59 (dd, J = 16.9, 7.2 Hz, 2H), 7.55 – 7.51 (m, 1H), 7.40 – 7.30 (m, 3H). ^{13}C NMR (126 MHz, $CDCl_3$) δ 133.84, 133.12, 132.61, 130.24, 128.81, 128.02, 127.73, 127.45, 118.88, 101.90.

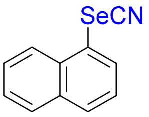

*1-selenocyanatonaphthalene (**3t**)*[19]: Prepared following general procedure and the reaction mixture was purified by flash column chromatography to afford the product **3t**(31.7 mg, 68%

yield). White solid. ^{1}H NMR (500 MHz, $CDCl_3$) δ 8.09 (d, J = 8.4 Hz, 1H), 7.92 (d, J = 7.3 Hz, 1H), 7.88 (d, J = 8.2 Hz, 1H), 7.81 (d, J = 8.1 Hz, 1H), 7.59 (t, J = 7.7 Hz, 1H), 7.51 (t, J = 7.5 Hz, 1H), 7.37 (t, J = 7.7 Hz, 1H). ^{13}C NMR (126 MHz, $CDCl_3$) δ 133.61, 133.35, 132.06, 130.68, 127.96, 127.13, 126.10, 125.53, 125.08, 119.42, 100.20.

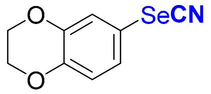

6-selenocyanato-2,3-dihydrobenzo[b][1,4]dioxine (**3u**): Prepared following general procedure and the reaction mixture was purified by flash column chromatography to afford the product **3u**(34.2 mg, 71% yield). White solid. ^{1}H NMR (500 MHz, $CDCl_3$) δ 7.12 (s, 1H), 7.09 – 7.01 (m, 1H), 6.80 (dd, J = 8.4, 2.5 Hz, 1H), 4.20 (s, 4H). ^{13}C NMR (126 MHz, $CDCl_3$) δ 144.63, 143.60, 126.39, 122.18, 118.05, 110.42, 100.91, 63.34, 63.19. HRMS: calculated for $C_9H_7NO_2SeNa$ $[M+Na]^+$: 263.9534, found: 263.9536

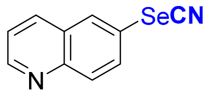

6-selenocyanatoquinoline (**3v**): Prepared following general procedure and the reaction mixture was purified by flash column chromatography to afford the product **3v**(29.5 mg, 63% yield). Yellow solid. ^{1}H NMR (500 MHz, $CDCl_3$) δ 8.82 (d, J = 3.9 Hz, 1H), 7.99 – 7.92 (m, 3H), 7.87 (d, J = 8.7 Hz, 1H), 7.33 (dd, J = 8.4, 4.2 Hz, 1H). ^{13}C NMR (126 MHz, $CDCl_3$) δ 149.81, 146.70, 134.50, 131.63, 129.41, 129.20, 127.88, 127.53, 124.21, 120.74. HRMS: calculated for $C_{10}H_6N_2SeNa$ $[M+Na]^+$: 256.9588, found: 256.9593.

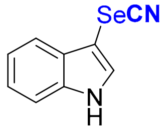

3-selenocyanato-1H-indole (**3w**): Prepared following general procedure and the reaction mixture was purified by flash column chromatography to afford the product **3w**(27.5 mg, 62% yield). Brown solid. ^{1}H NMR (500 MHz, $CDCl_3$) δ 8.67 (s, 1H), 7.66 – 7.57 (m, 1H), 7.28 – 7.24 (m, 2H), 7.20 – 7.16 (m, 2H). ^{13}C NMR (126 MHz, $CDCl_3$) δ 134.97, 130.93, 127.61, 122.60, 120.70, 118.36, 110.97, 101.30, 87.99. HRMS: calculated for $C_9H_6N_2SeNa$ $[M+Na]^+$: 244.9588, found: 244.9590.

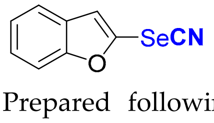

2-selenocyanatobenzofuran (**3x**)[19]: Prepared following general procedure and the reaction mixture was purified by flash column chromatography to afford the product **3x**(28.5 mg, 64% yield). White solid. ^{1}H NMR (500 MHz, $CDCl_3$) δ 7.54 (d, J = 7.8 Hz, 1H), 7.48 (d, J = 8.8 Hz, 1H), 7.33 (t, J = 7.3 Hz, 1H), 7.23 (t, J = 7.5 Hz, 1H), 7.18 (s, 1H). ^{13}C NMR (126 MHz, $CDCl_3$) δ 156.87, 131.06, 126.49, 125.63, 122.79, 120.45, 117.88, 110.68, 97.34.

4. References

15. Zhang, X.; Huang, X.-B.; Zhou, Y.-B.; Liu, M.-C.; Wu, H.-Y. Metal-Free Synthesis of Aryl Selenocyanates and Selenaheterocycles with Elemental Selenium. *Chem.-Eur. J.* **2021**, *27*, 944–948.

16. Redon, S.; Kosso, A.R.O.; Broggi, J.; Vanelle, P. Metal-Free ipso-Selenocyanation of Arylboronic Acids Using Malononitrile and Selenium Dioxide. *Synthesis* **2019**, *51*, 3758–3764.

19. He, D.; Yao, J.; Ma, B.; Wei, J.; Hao, G.; Tuo, X.; Guo, S.; Fu, Z.; Cai, H. An electrochemical method for deborylative seleno/thiocyanation of arylboronic acids under catalyst- and oxidant-free conditions. *Green Chem.* **2020**, *22*, 1559–1564.

5. ^{1}H and ^{13}C NMR spectra of products

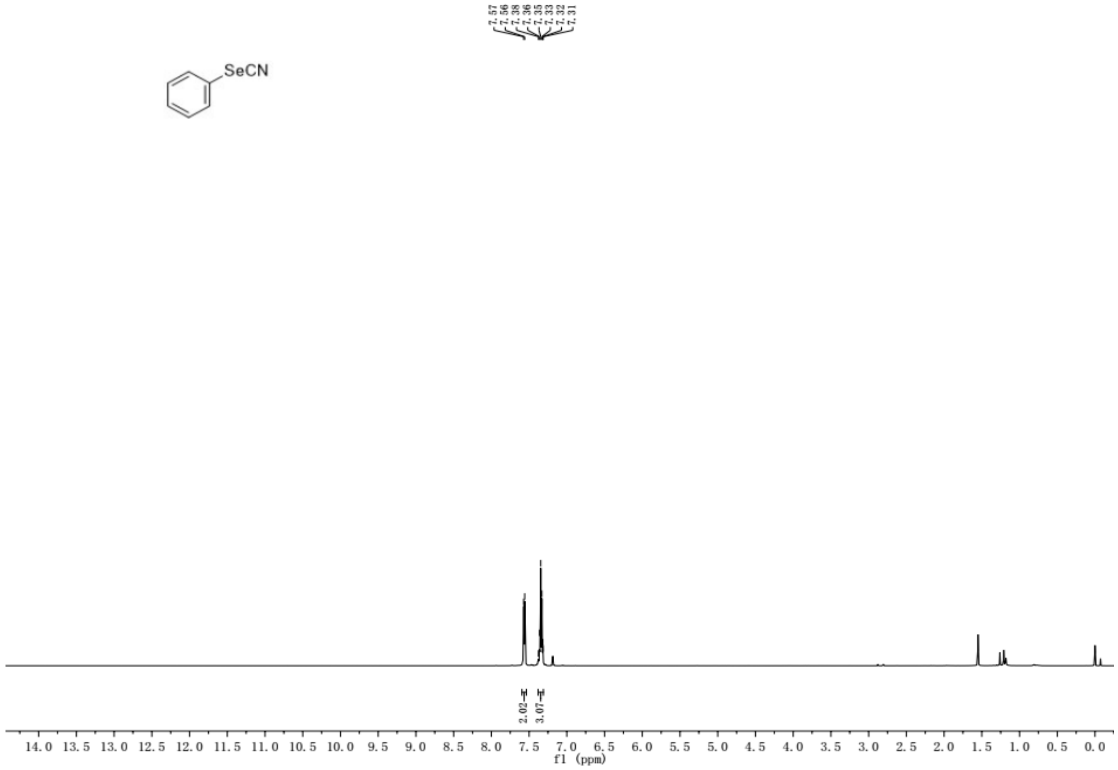

^{1}H NMR of compound **3a**

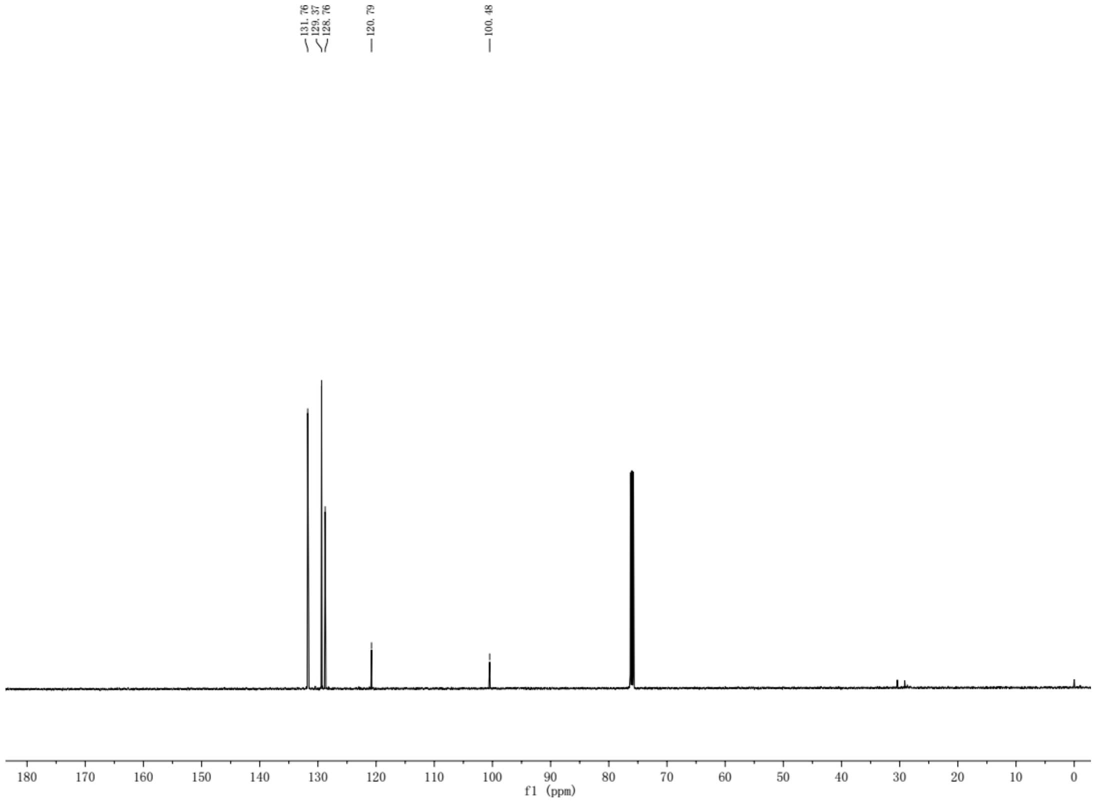

^{13}C NMR of compound **3a**

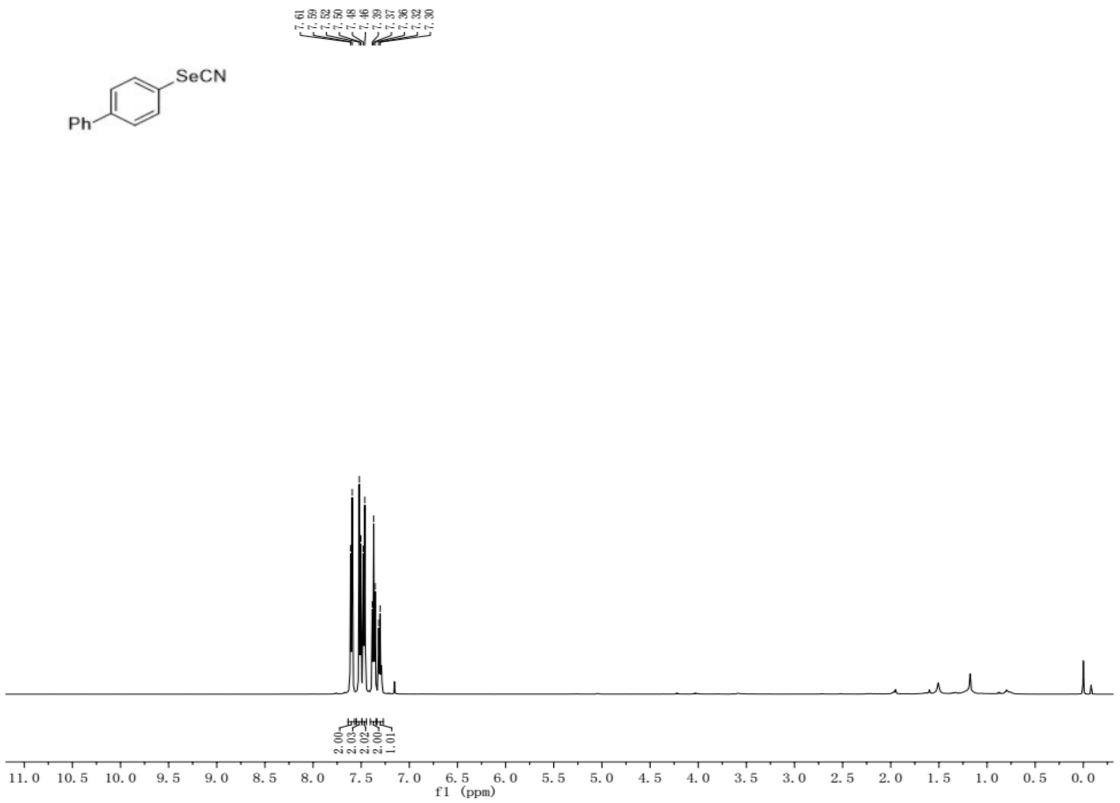

^{1}H NMR of compound **3b**

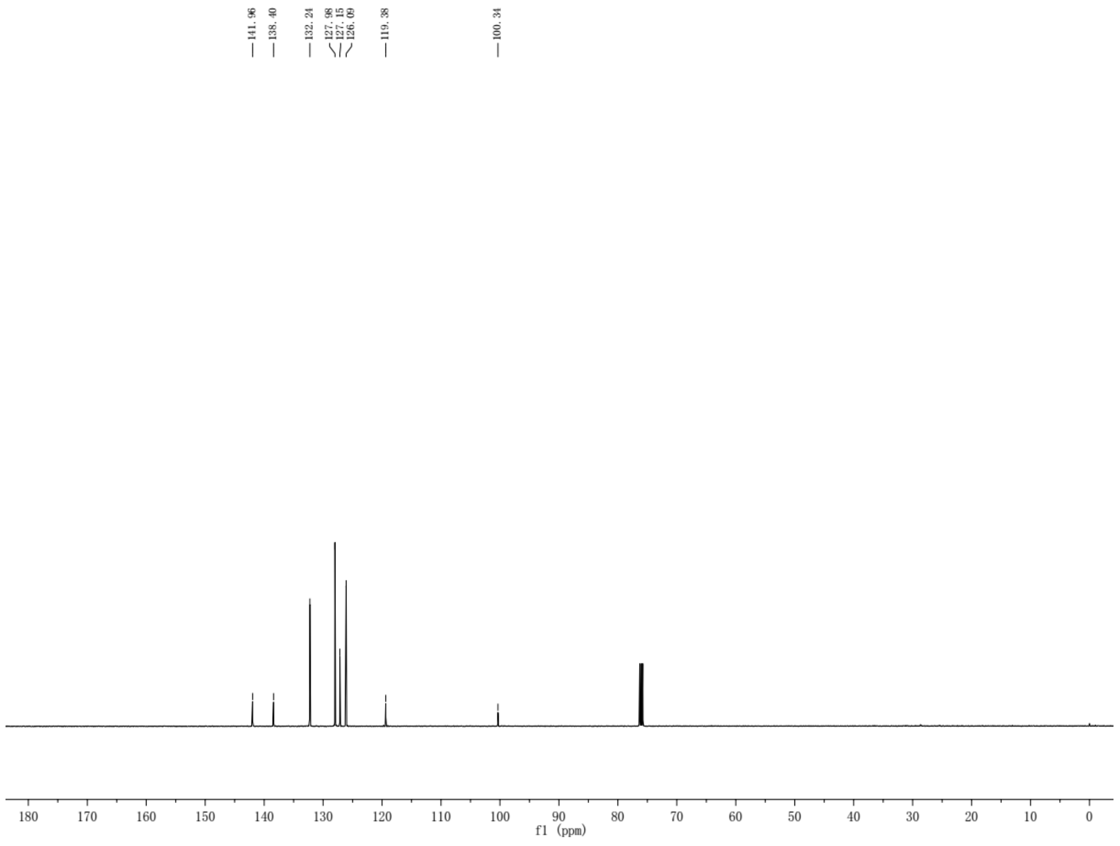

^{13}C NMR of compound **3b**

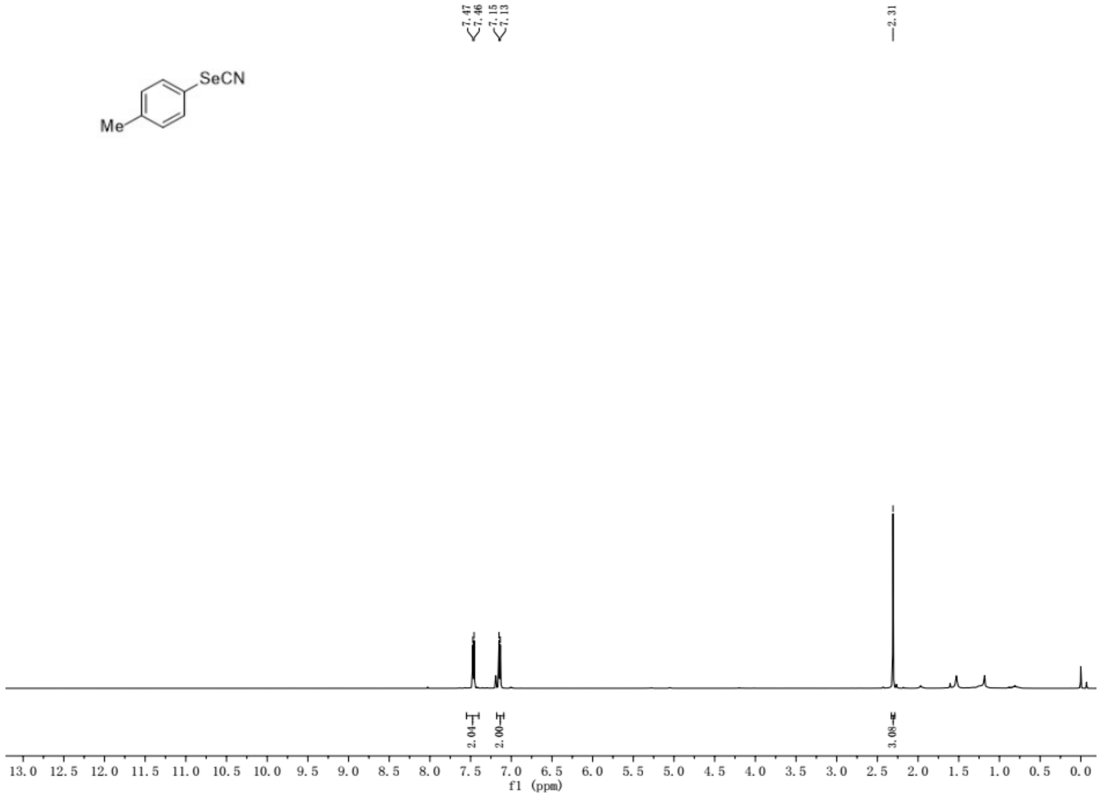

^{1}H NMR of compound **3c**

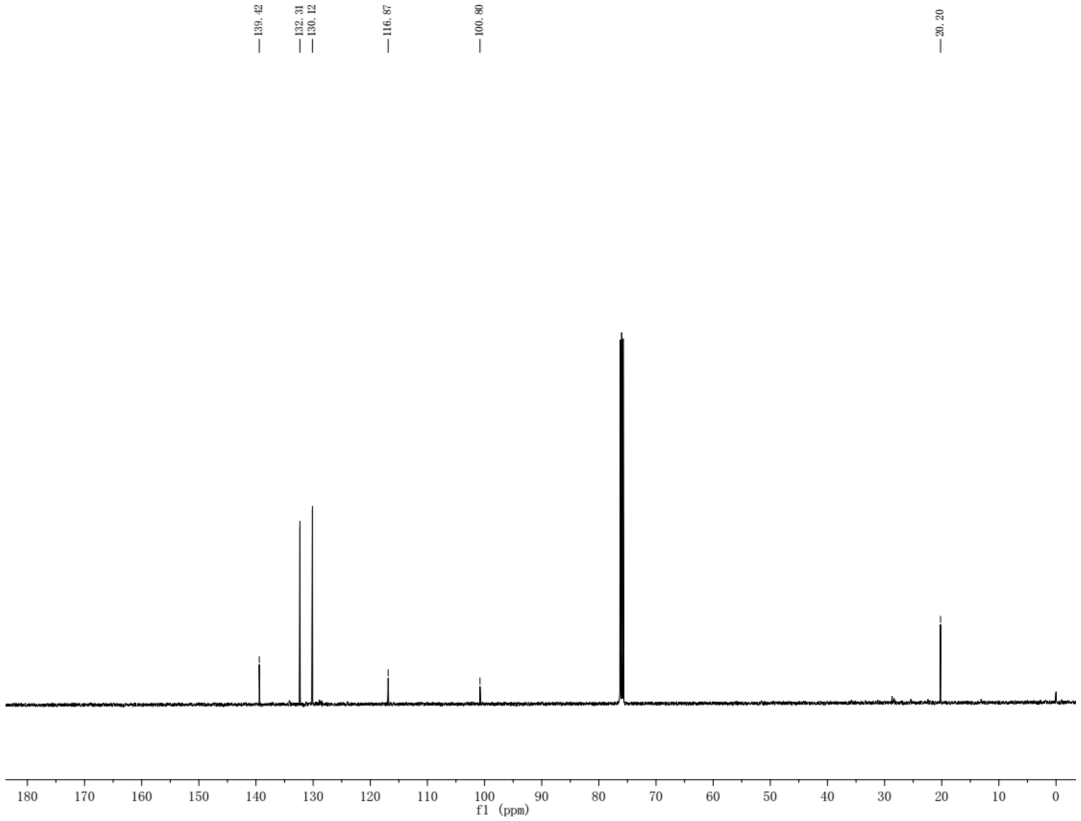

^{13}C NMR of compound **3c**

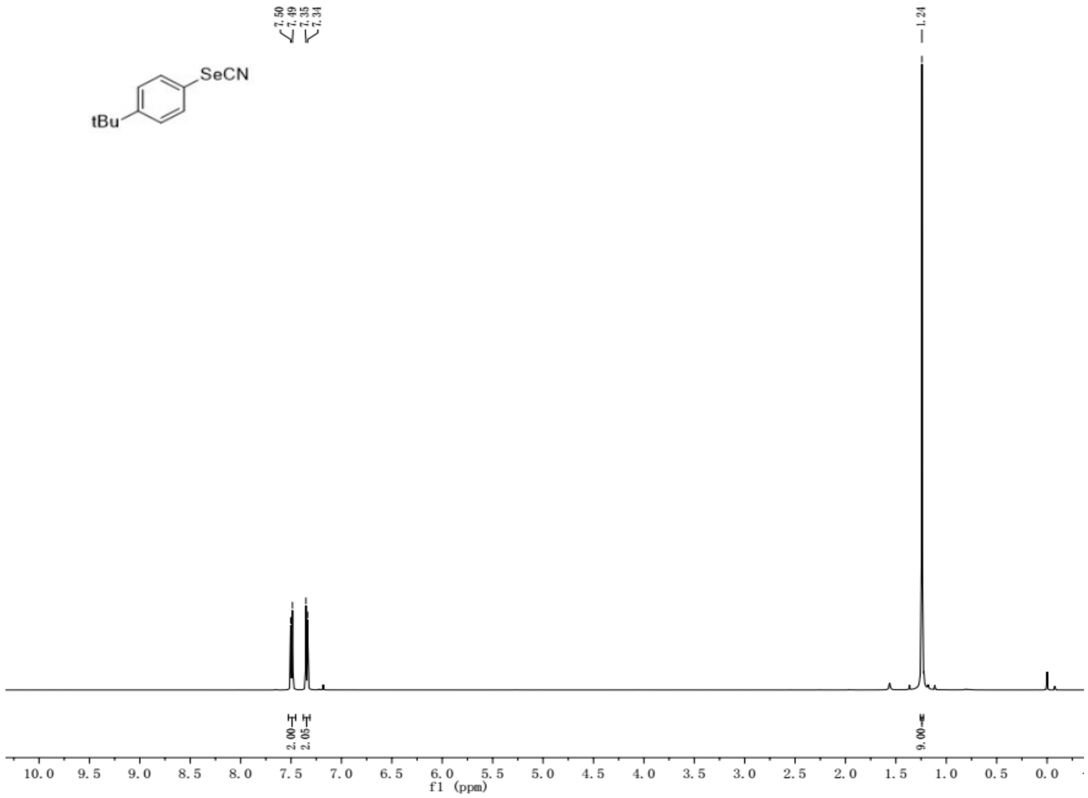

^{1}H NMR of compound **3d**

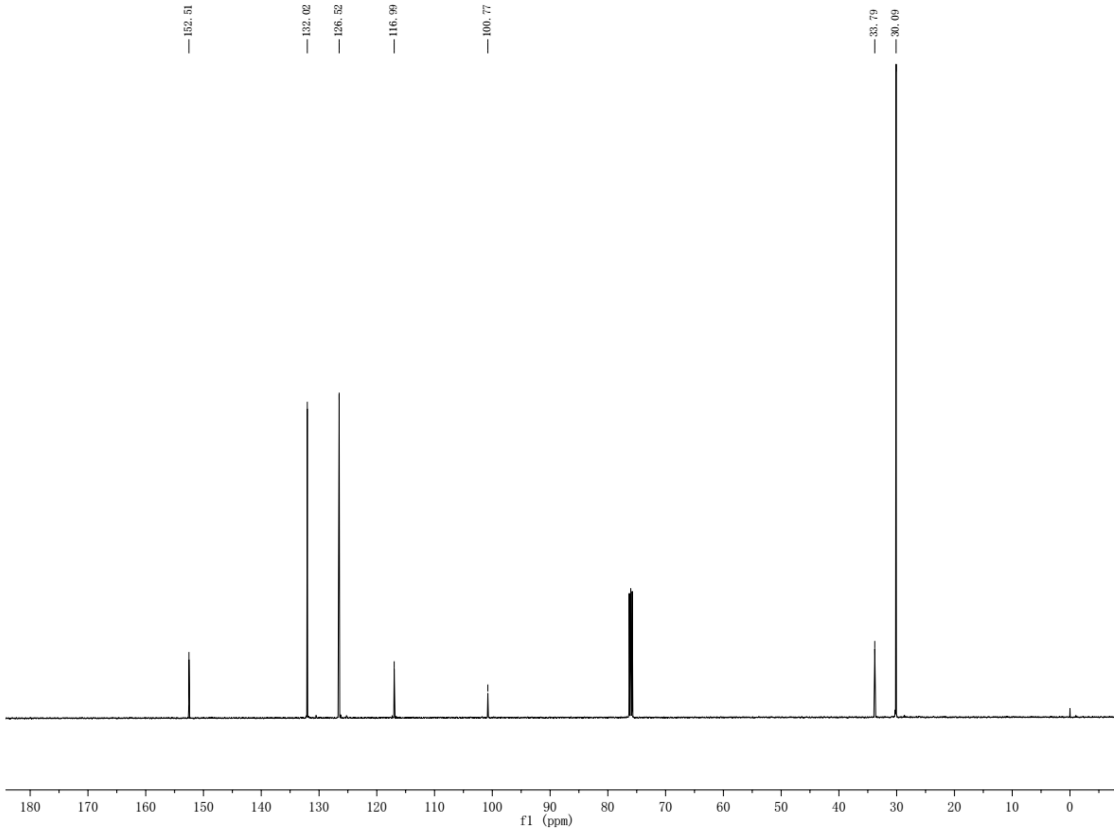

^{13}C NMR of compound **3d**

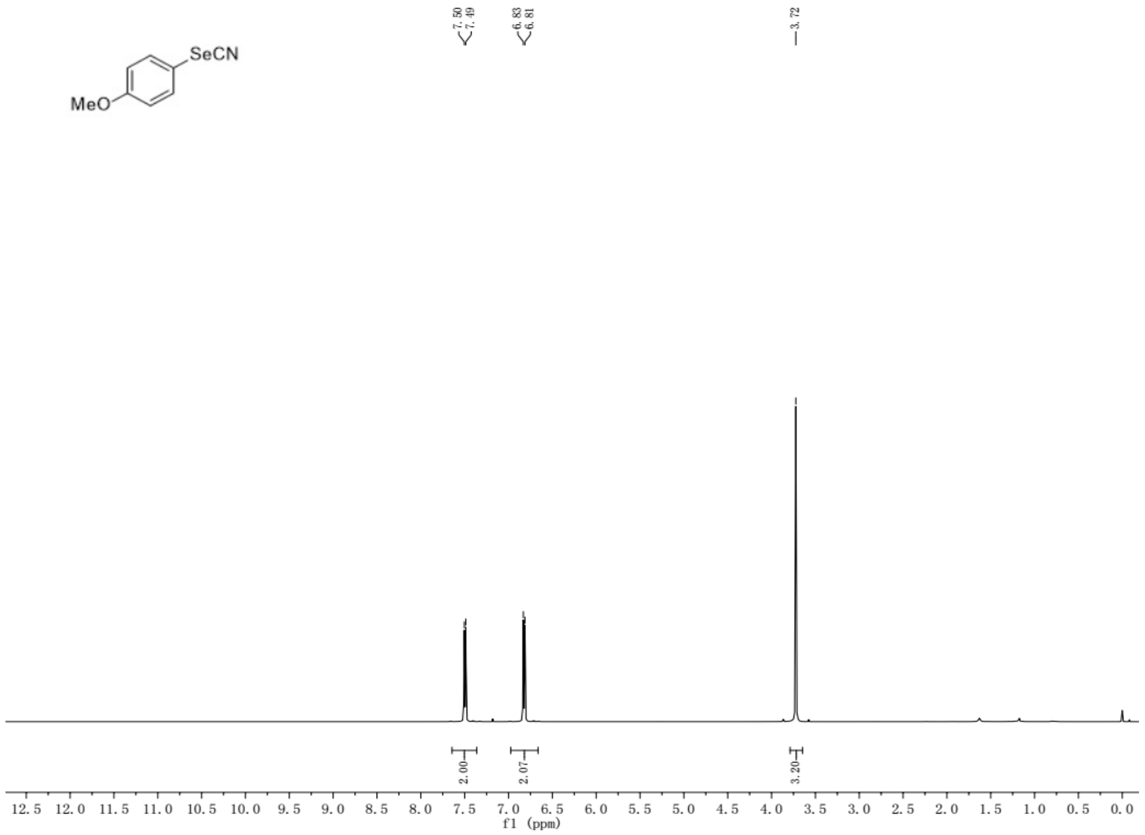

^{1}H NMR of compound **3e**

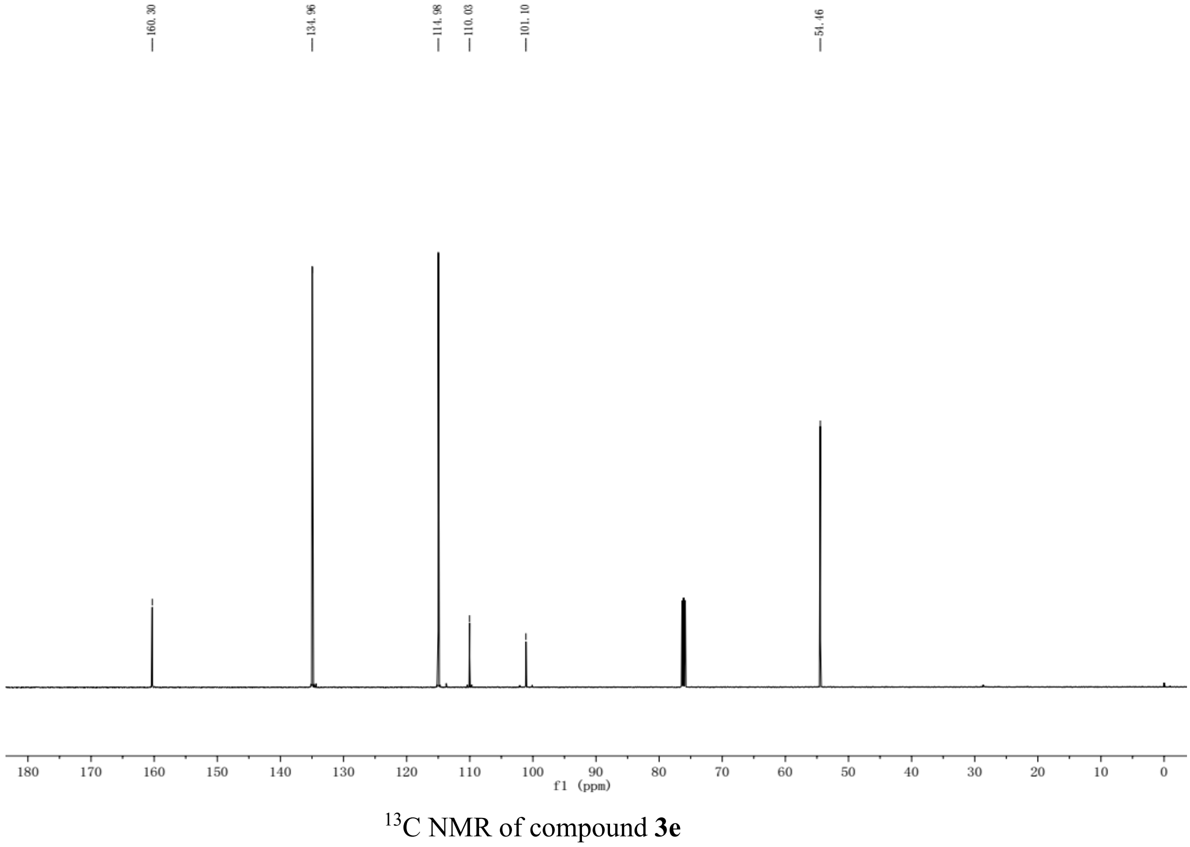

^{13}C NMR of compound **3e**

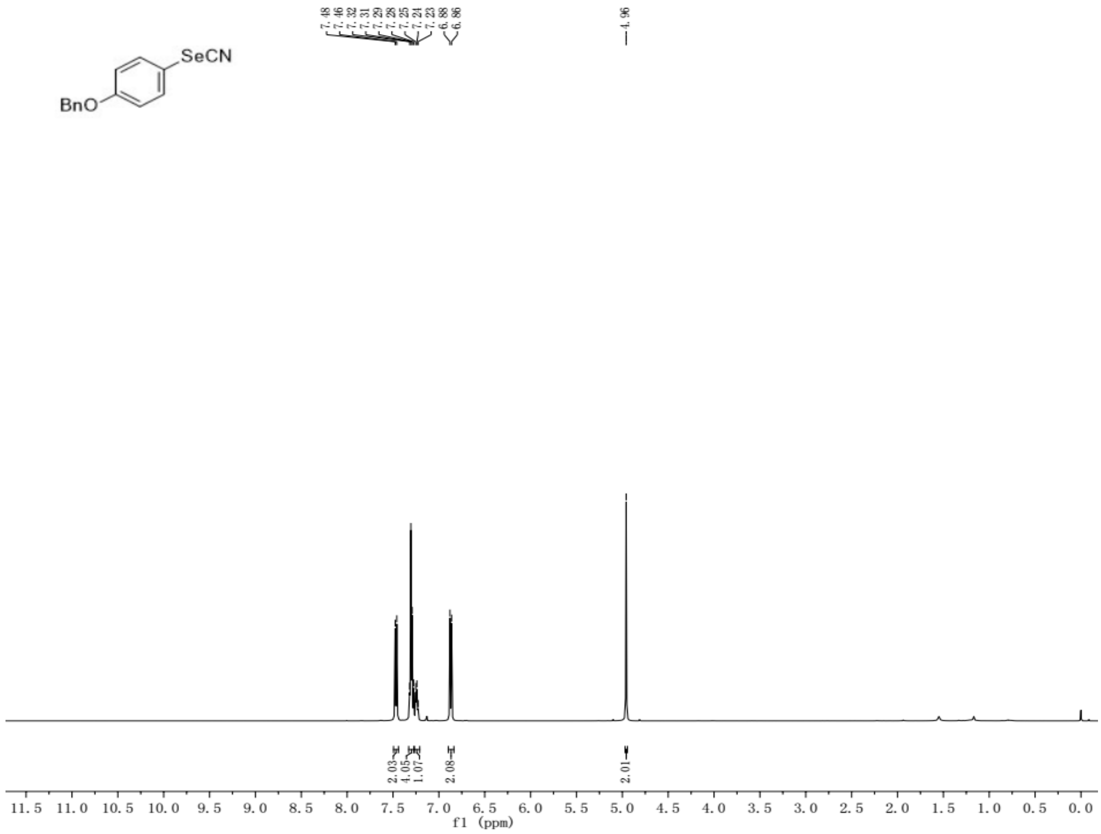

^{1}H NMR of compound **3f**

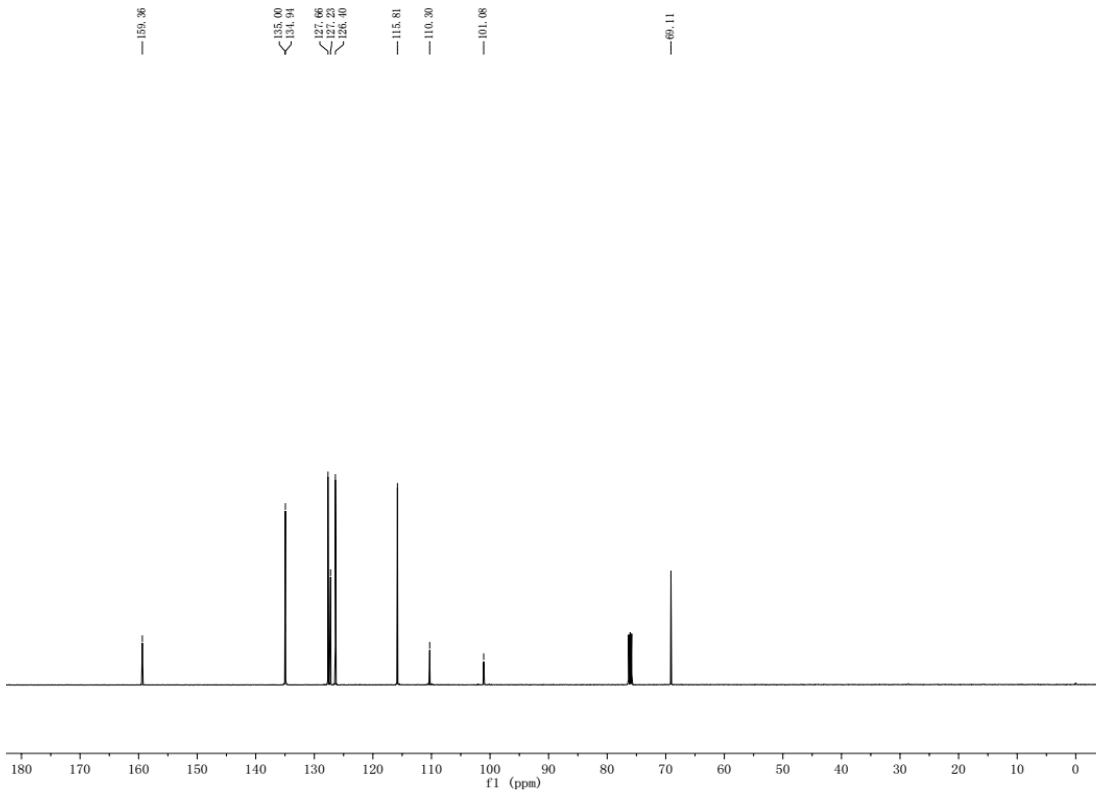

^{13}C NMR of compound **3f**

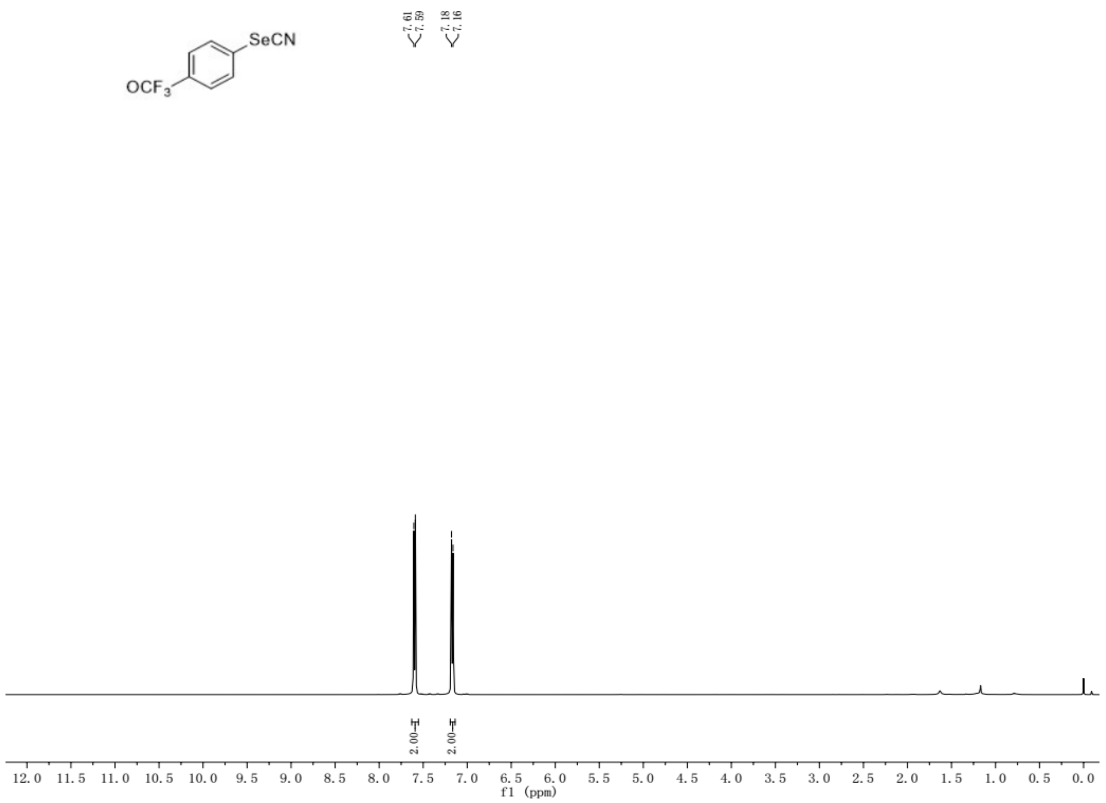

^{1}H NMR of compound **3g**

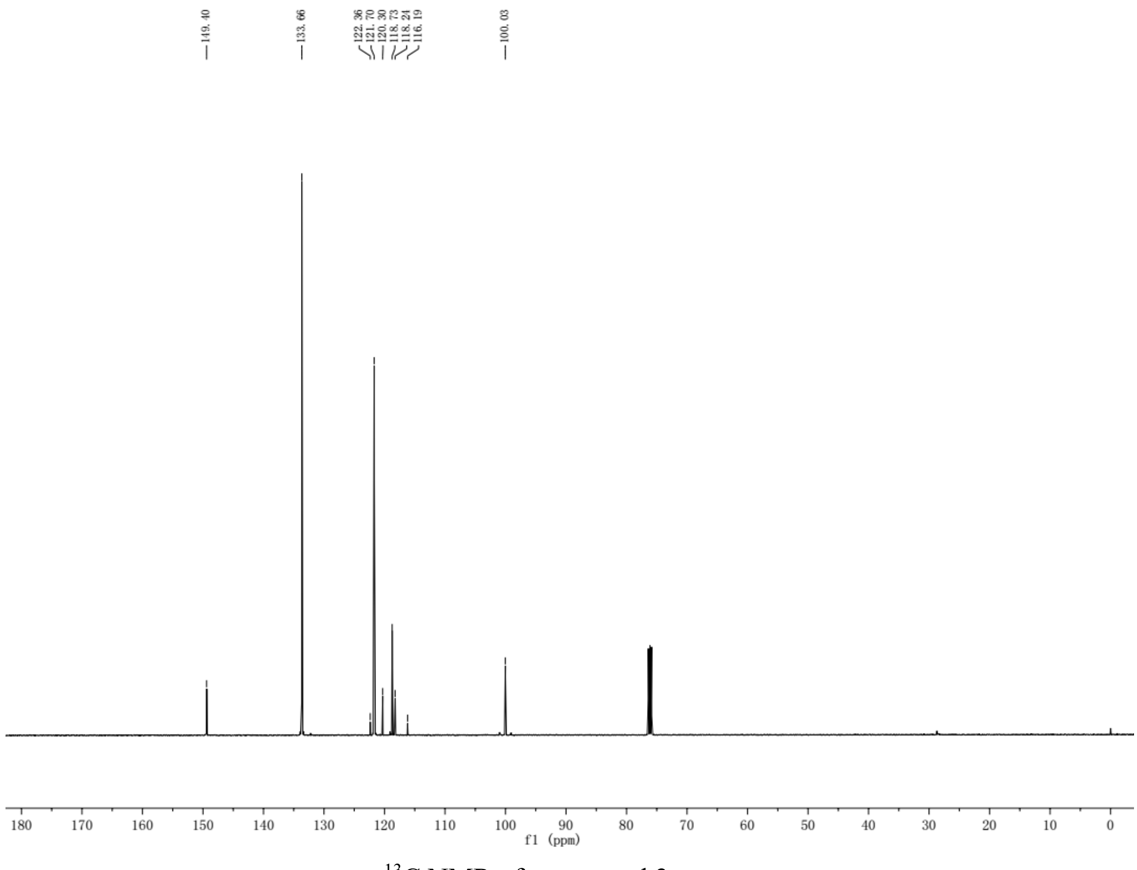

^{13}C NMR of compound **3g**

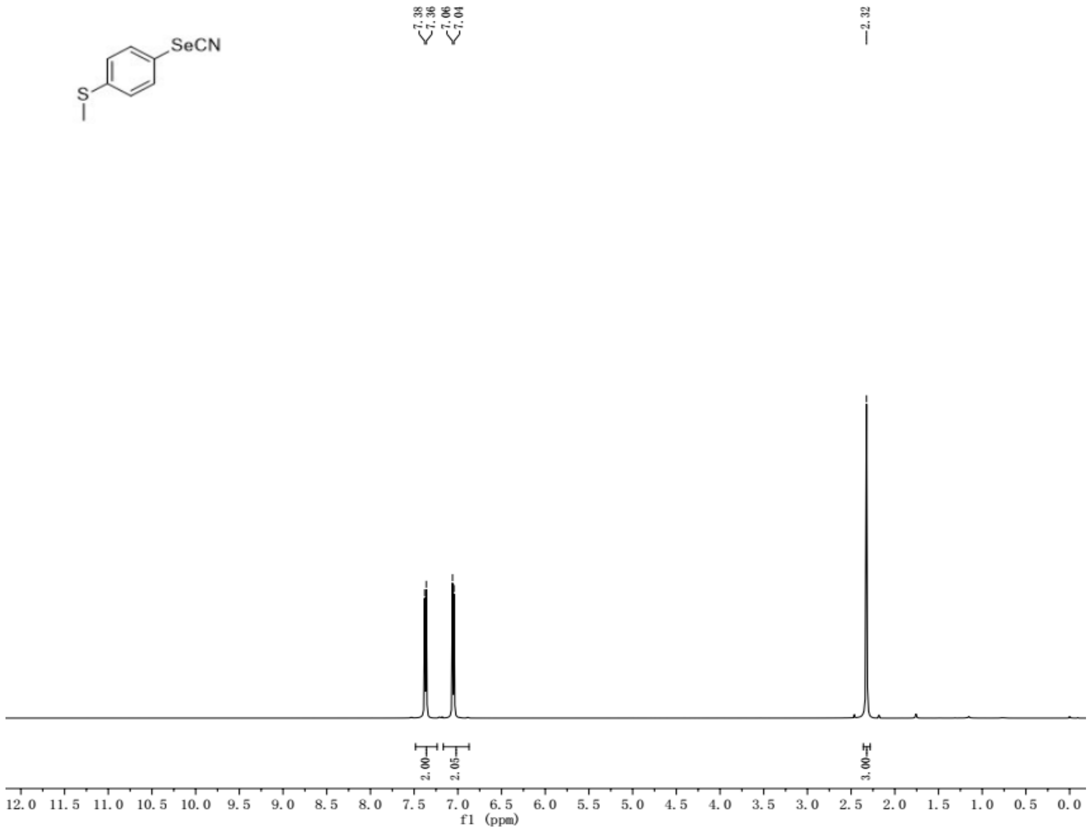

^{1}H NMR of compound **3h**

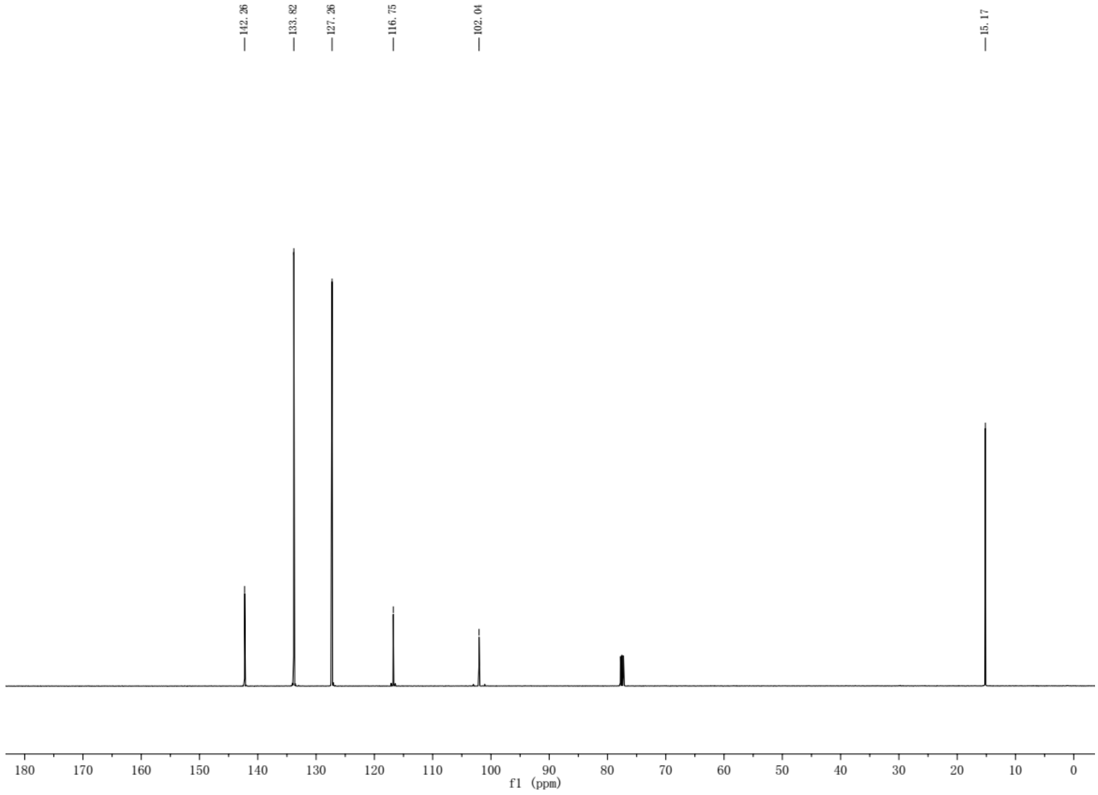

^{13}C NMR of compound **3h**

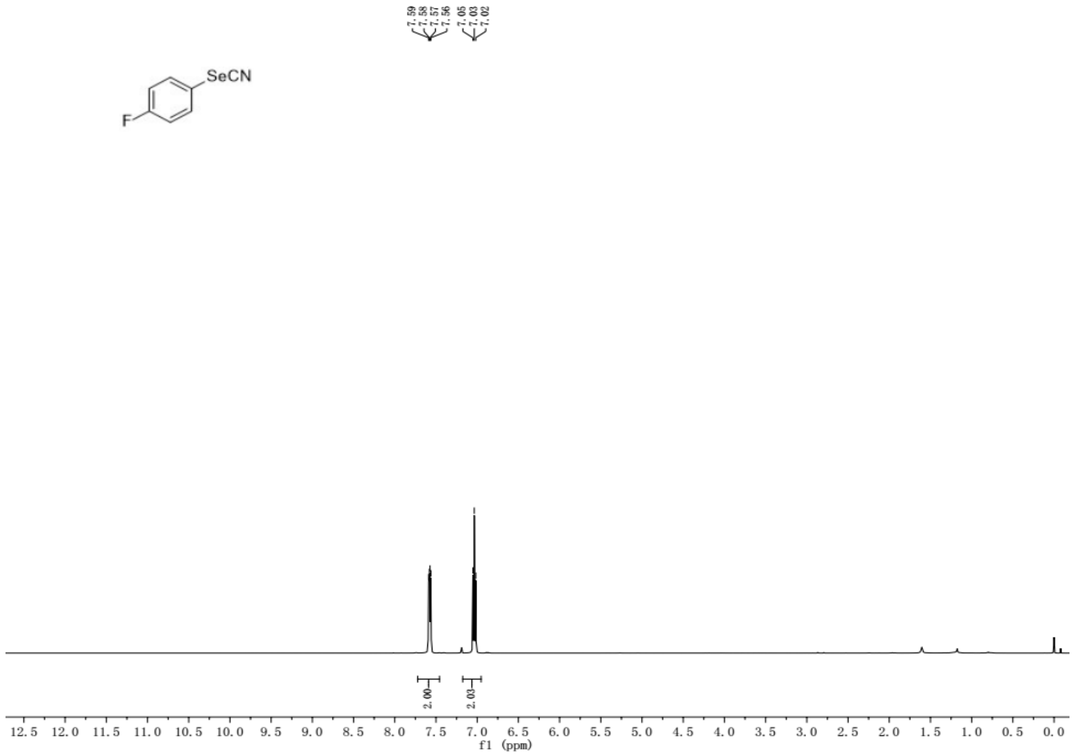

^{1}H NMR of compound **3i**

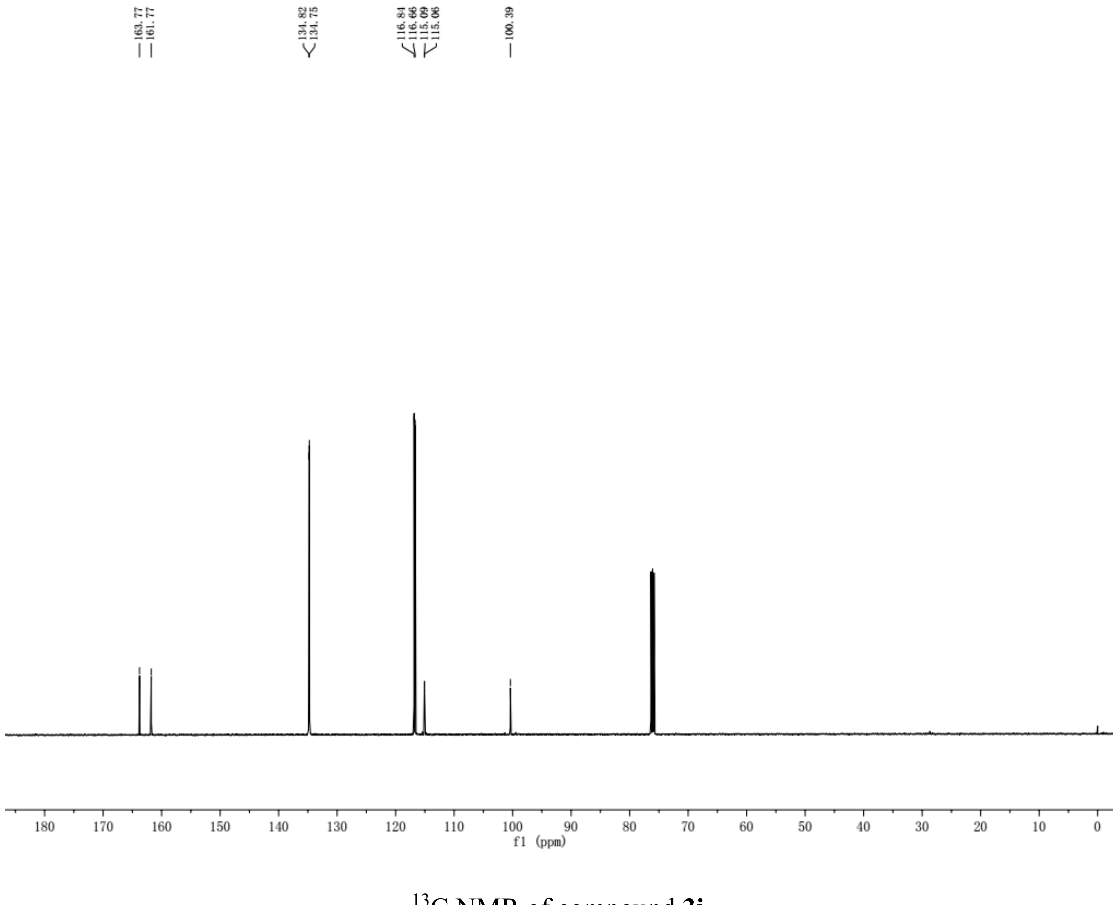

^{13}C NMR of compound **3i**

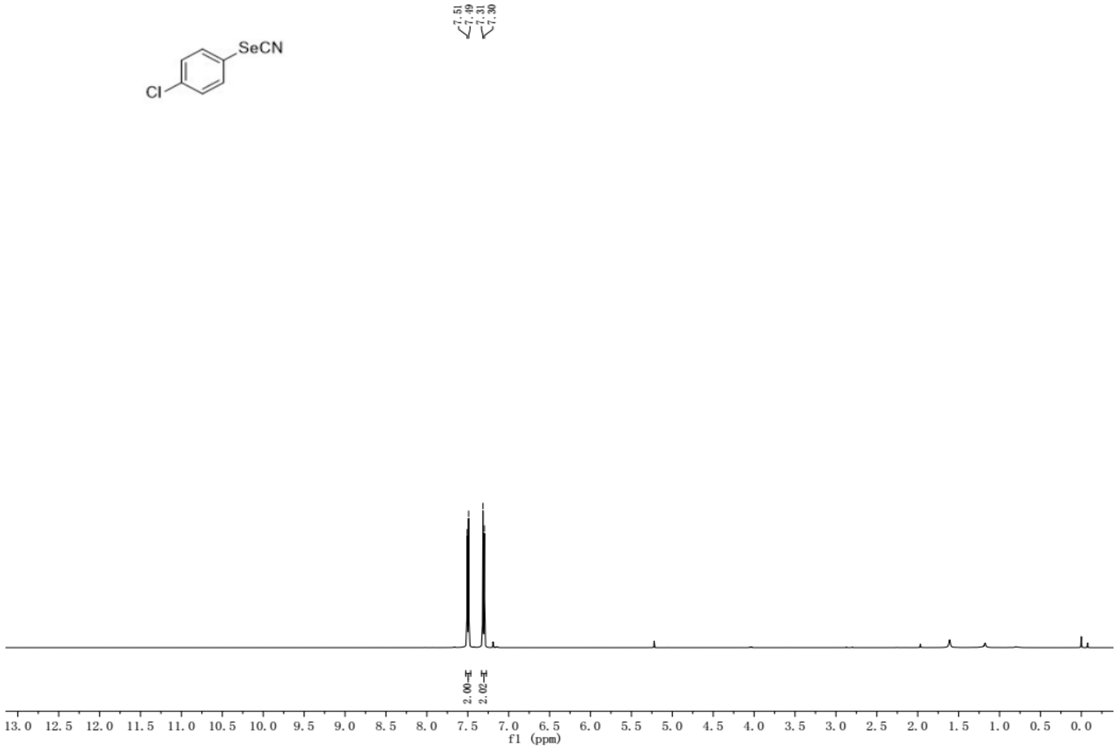

^{1}H NMR of compound **3j**

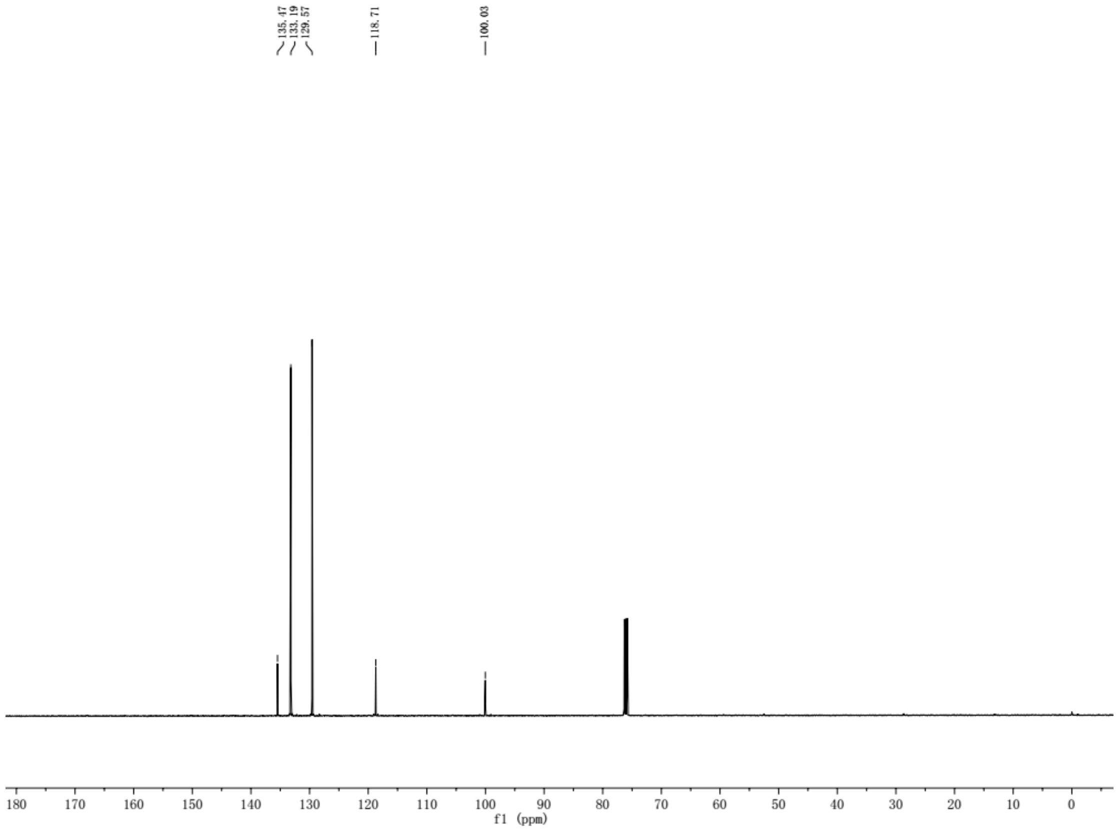

^{13}C NMR of compound **3j**

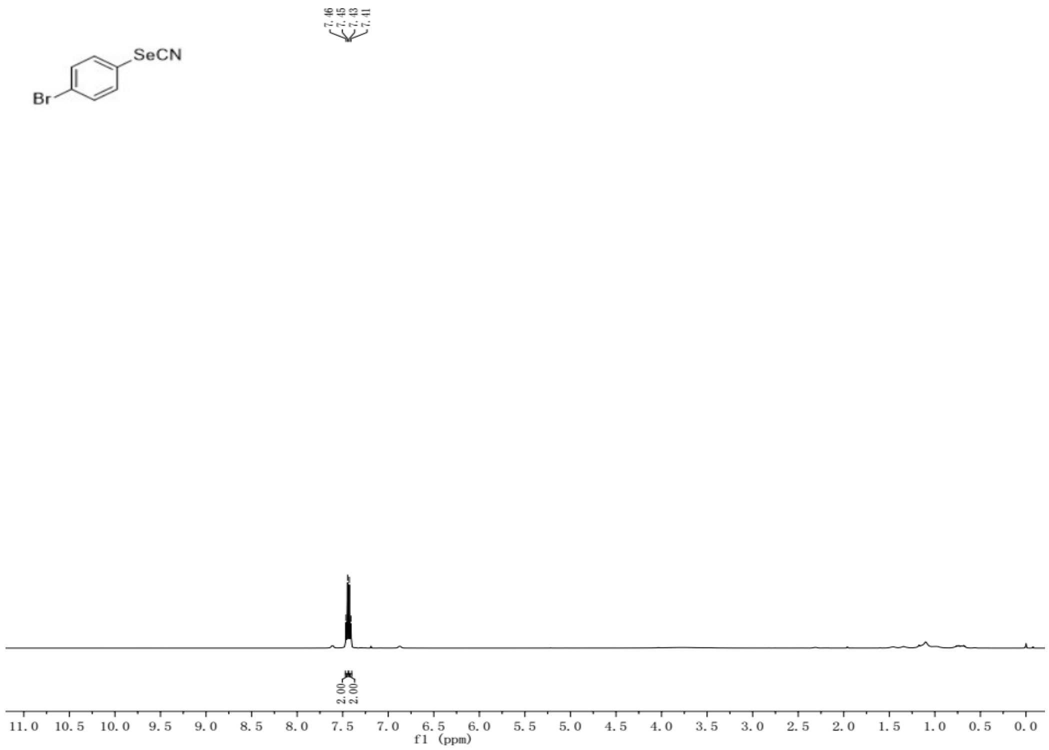

^{1}H NMR of compound **3k**

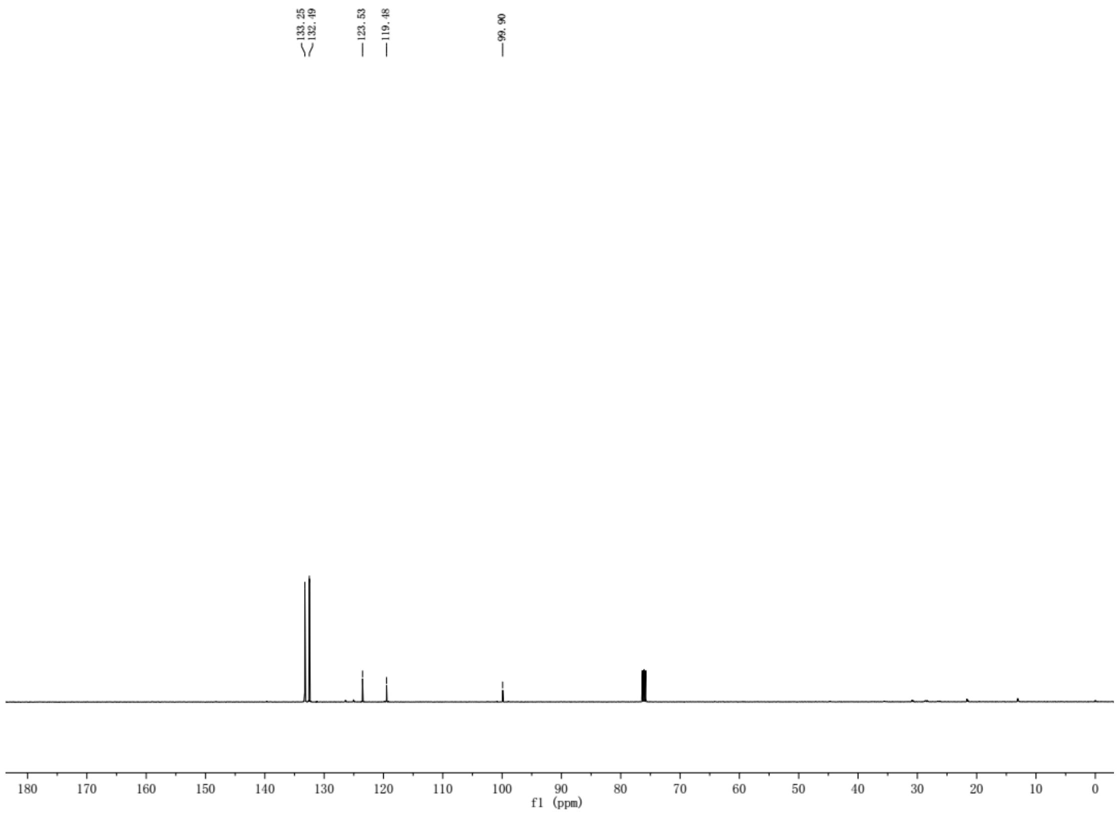

^{13}C NMR of compound **3k**

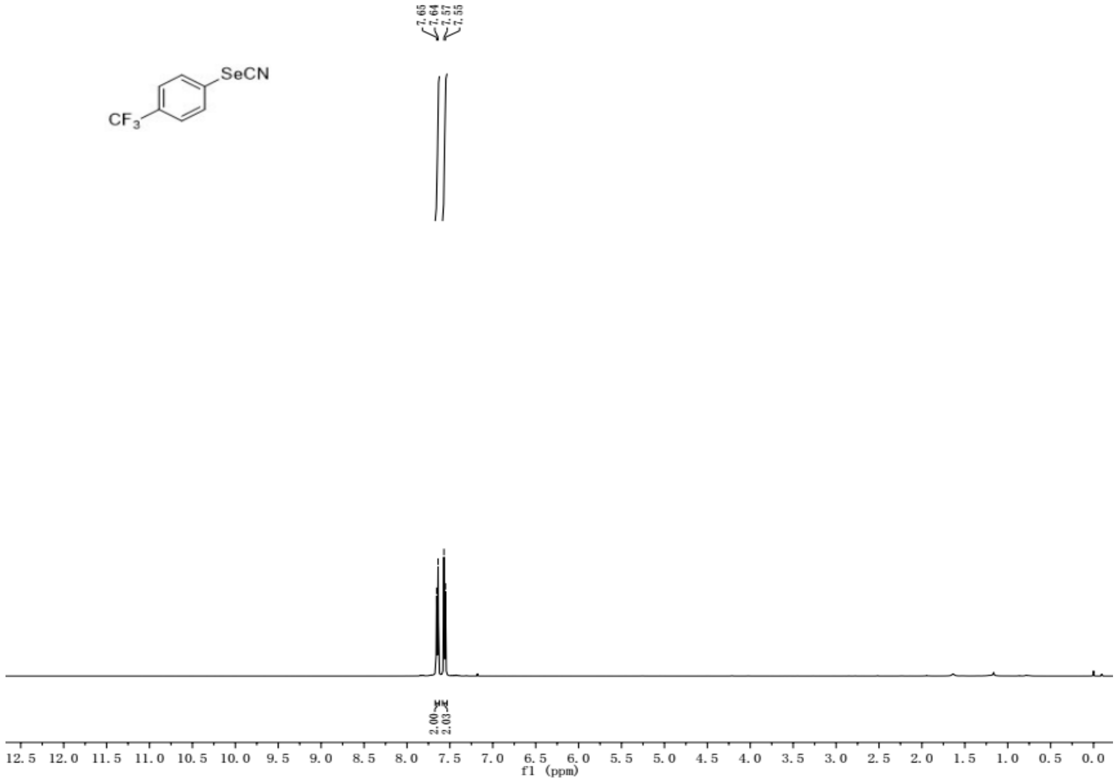

^{1}H NMR of compound **3l**

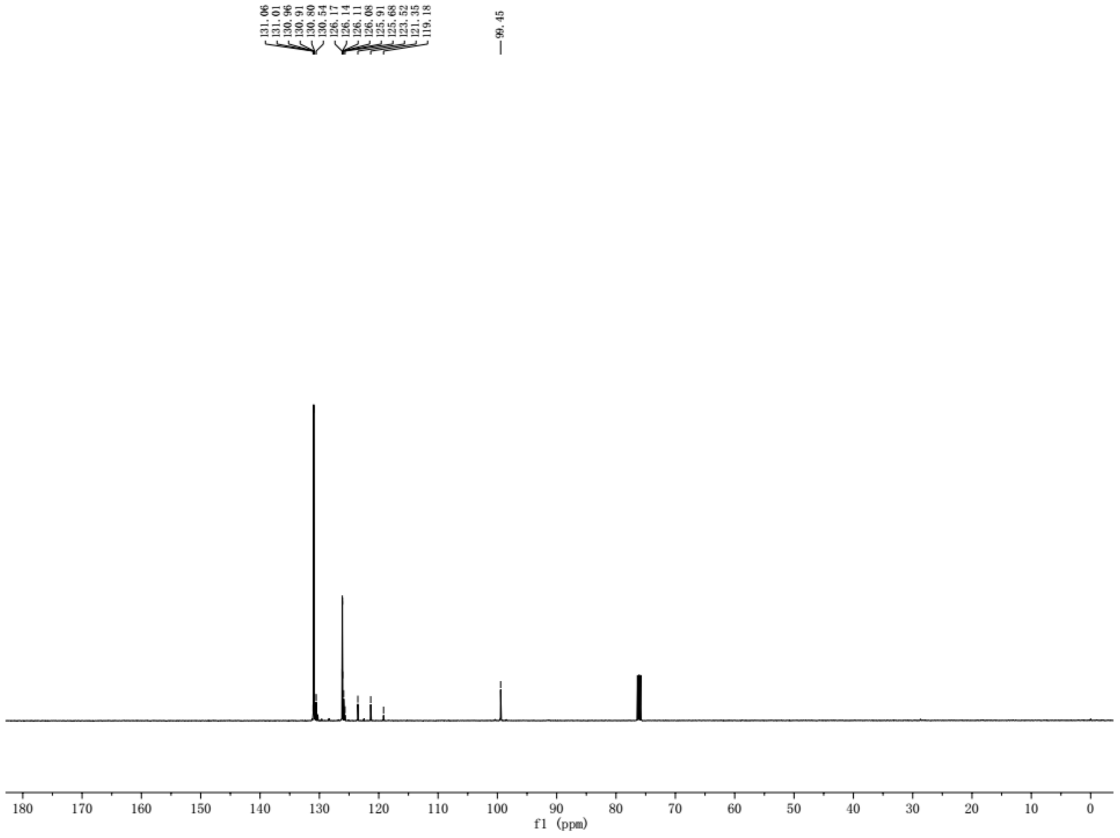

^{13}C NMR of compound **3l**

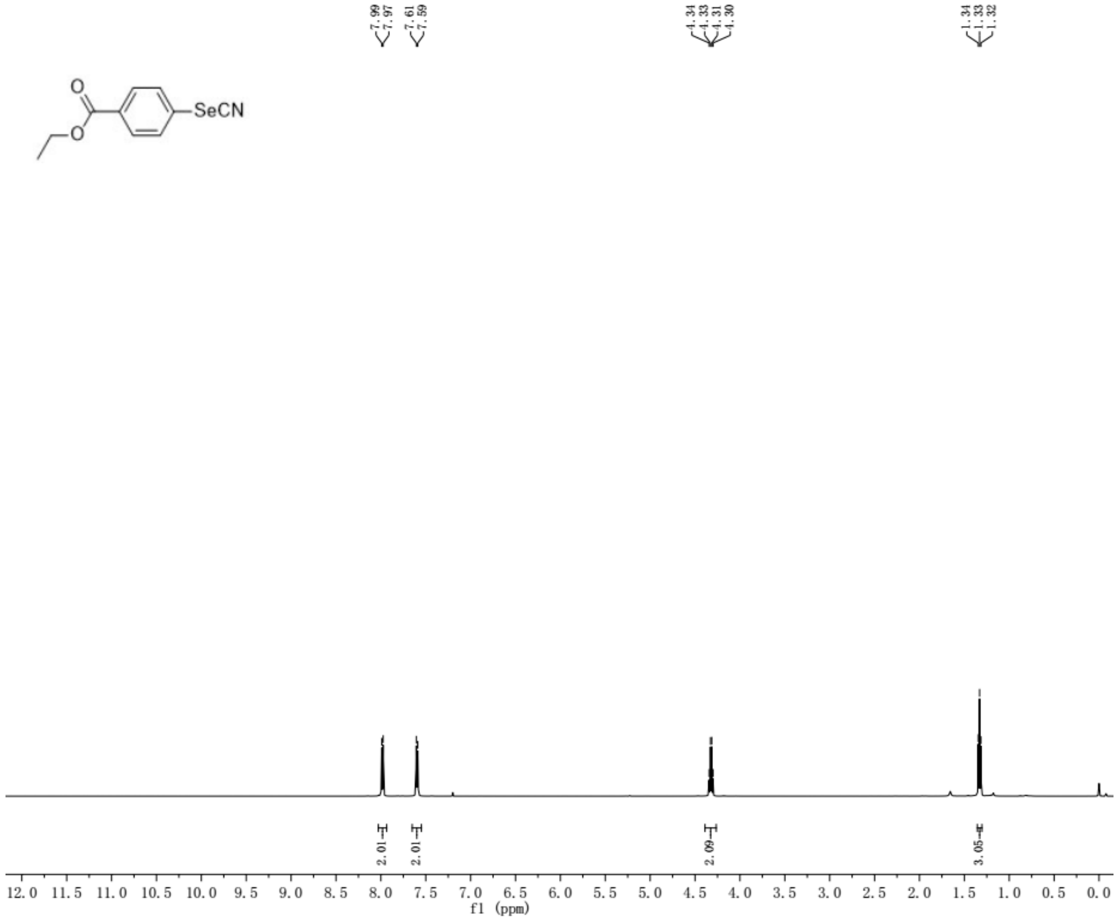

^{1}H NMR of compound **3m**

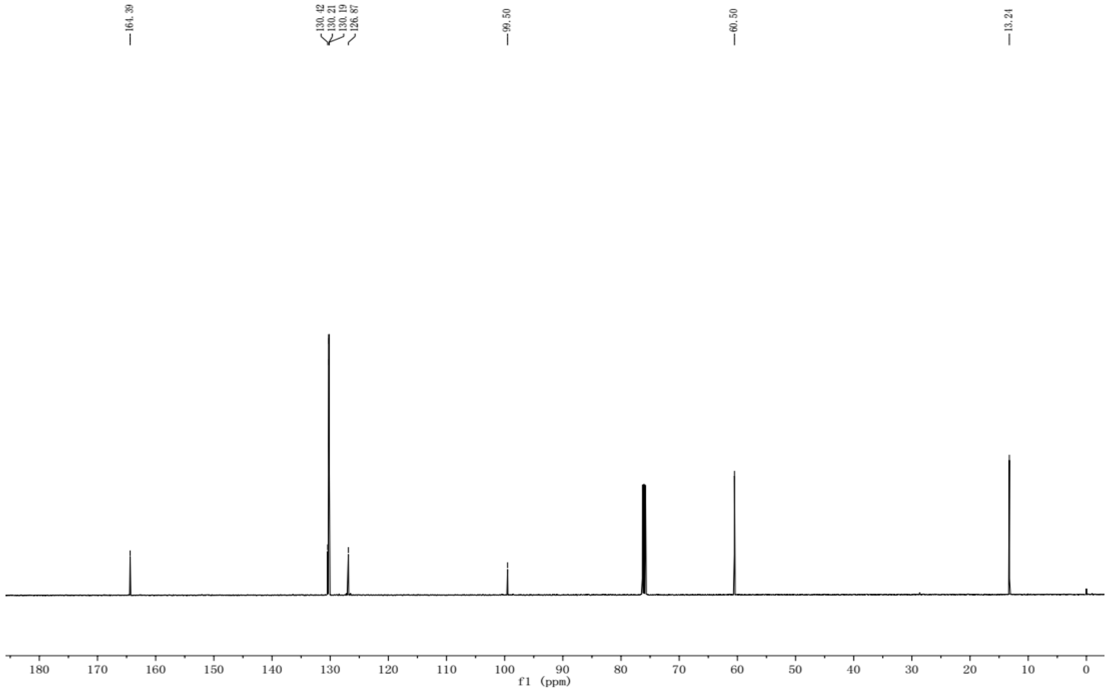

^{13}C NMR of compound **3m**

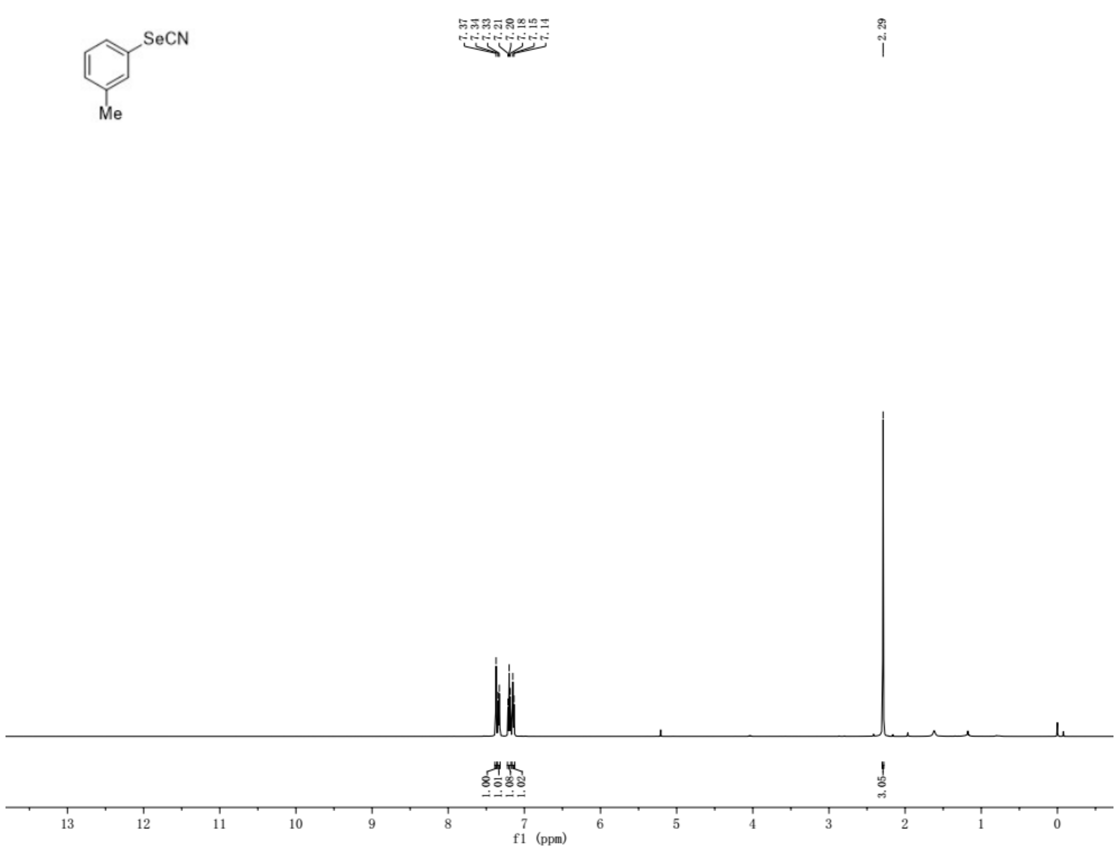

^{1}H NMR of compound **3n**

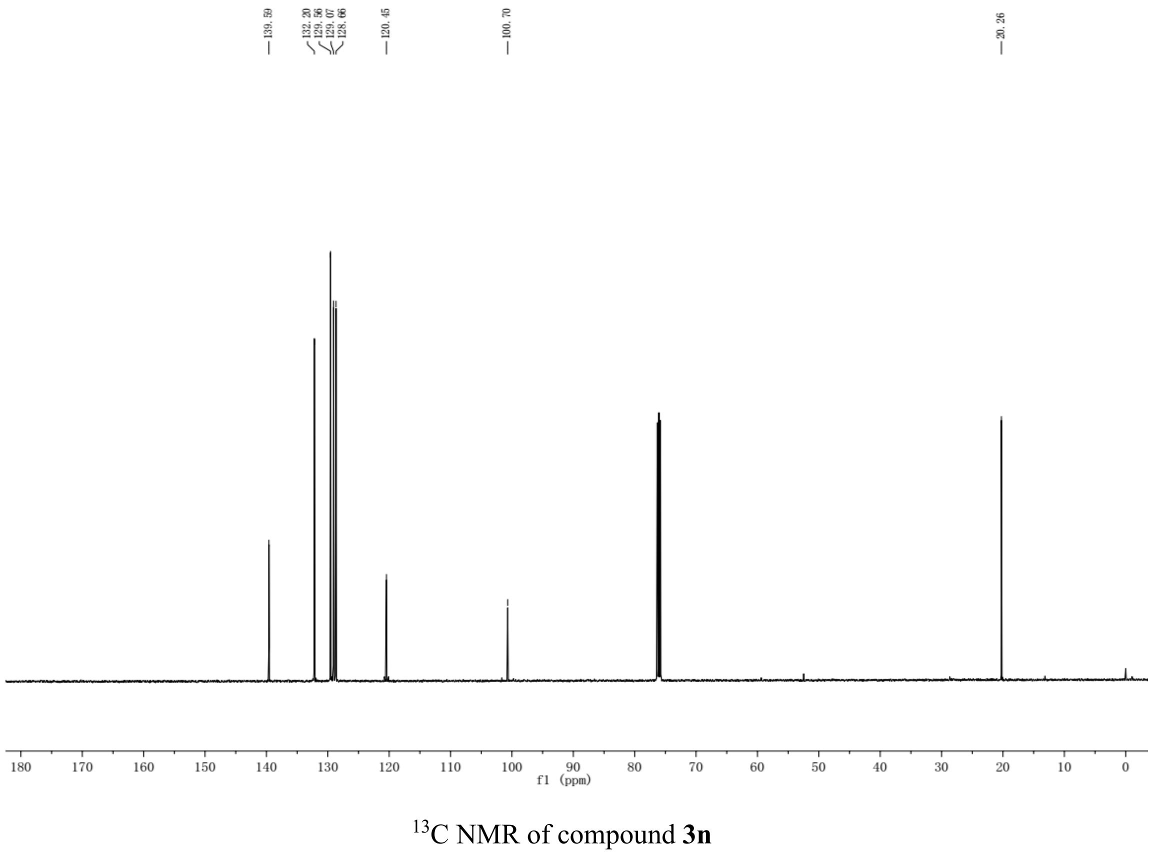

^{13}C NMR of compound **3n**

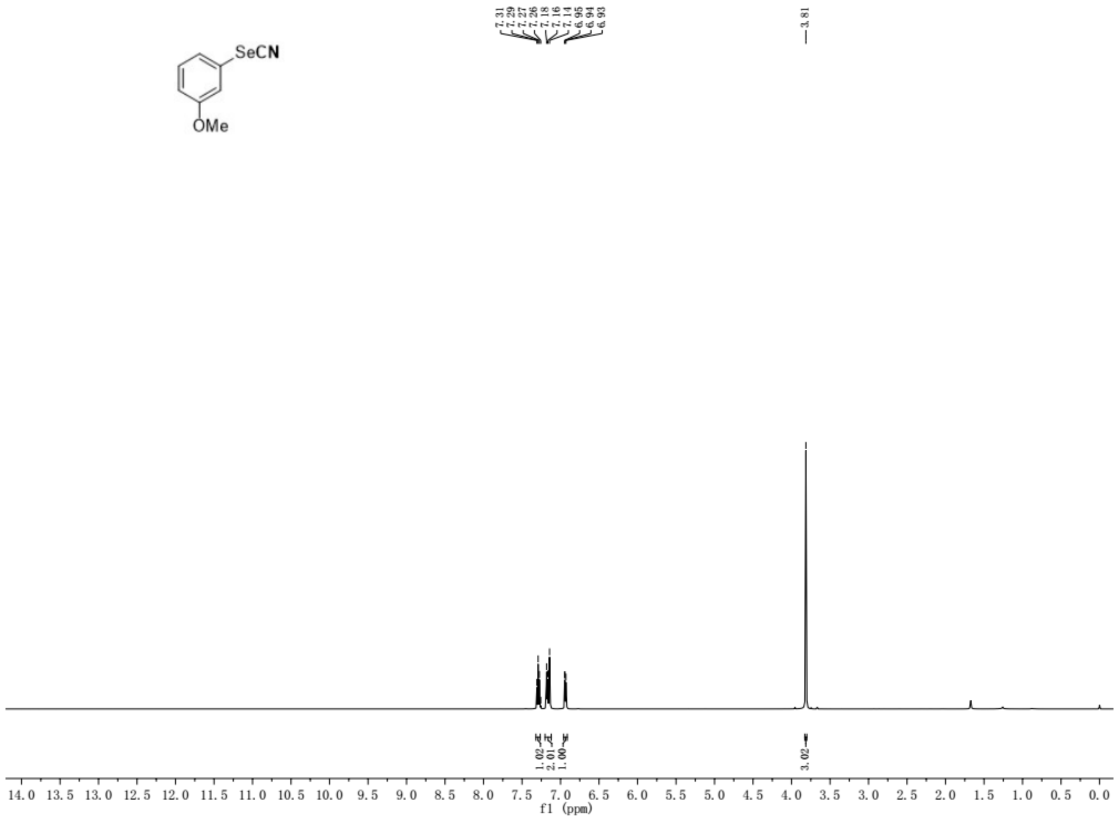

^{1}H NMR of compound **3o**

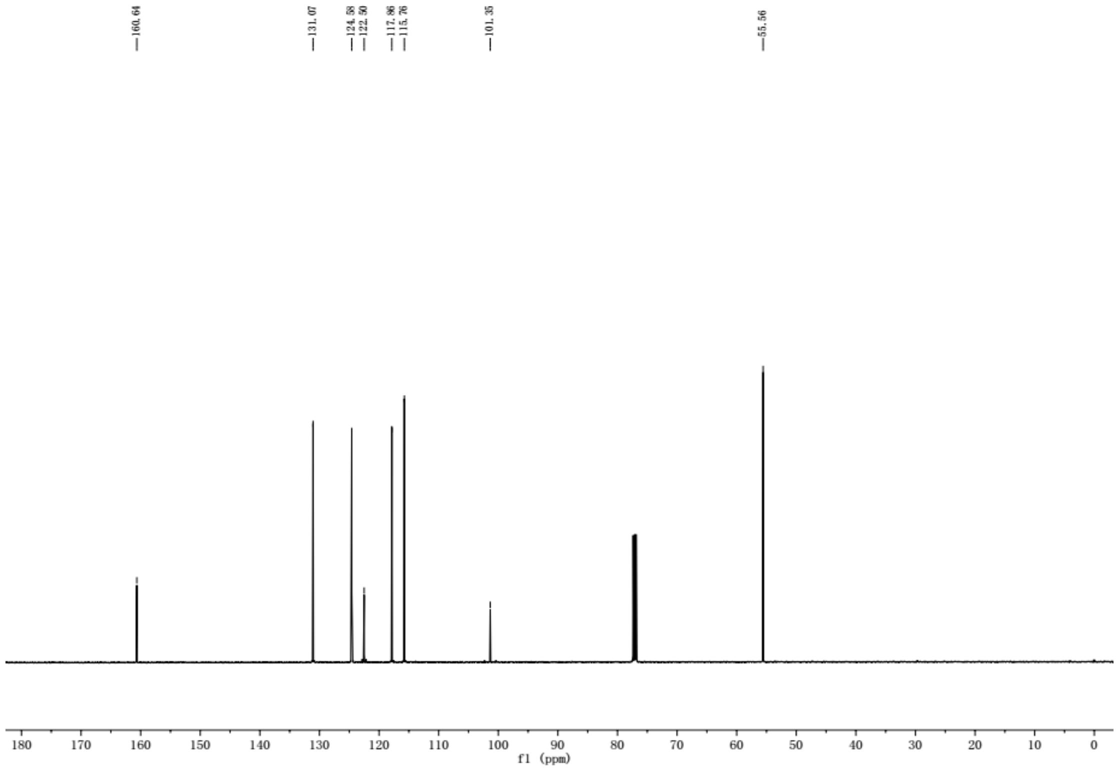

^{13}C NMR of compound **3o**

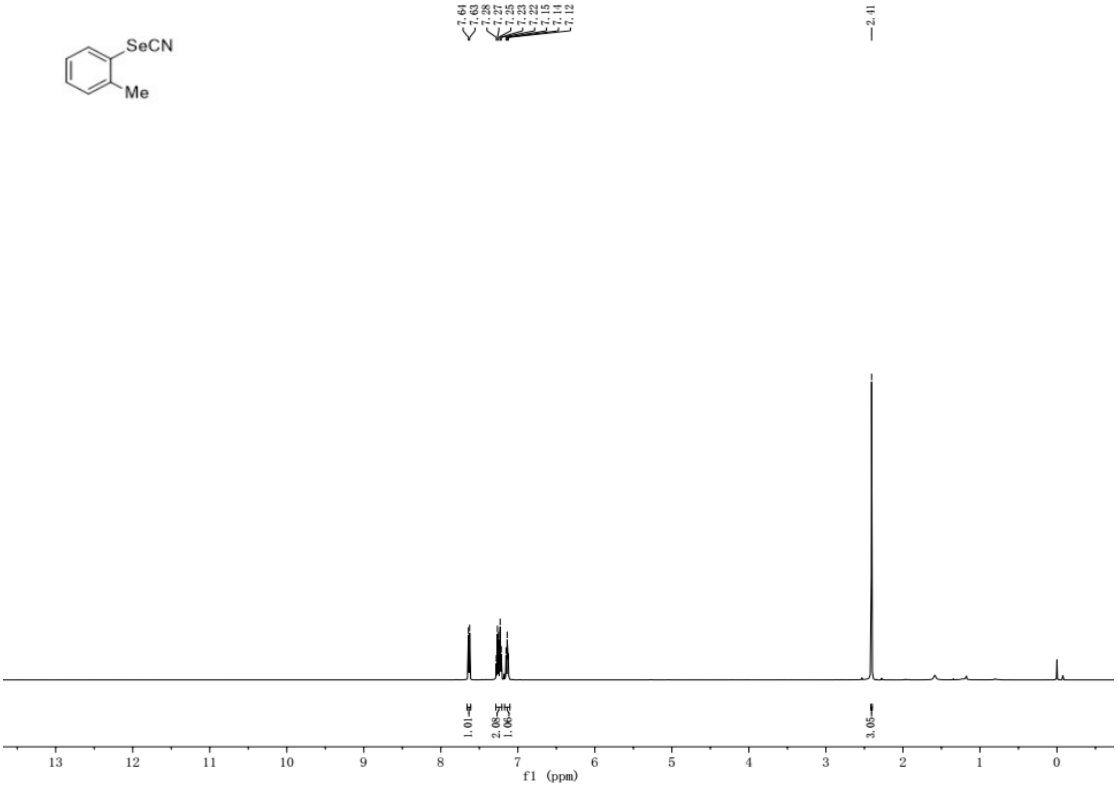

^{1}H NMR of compound **3p**

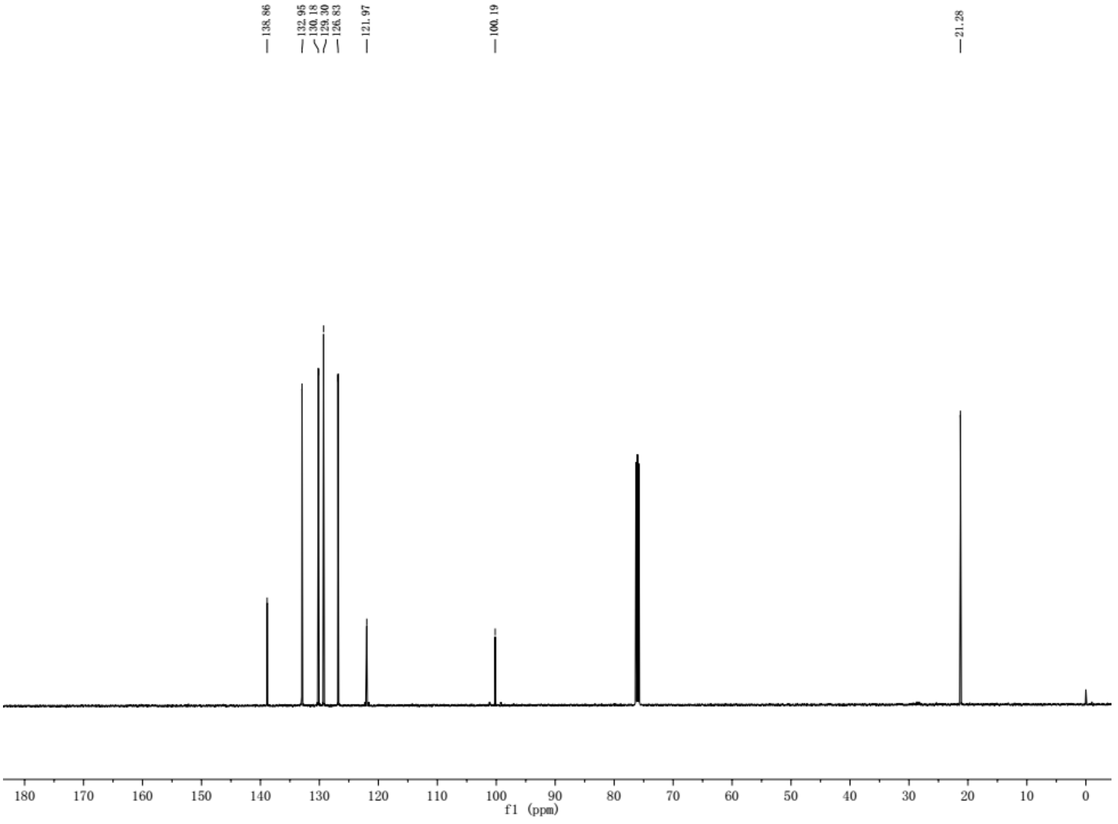

^{13}C NMR of compound **3p**

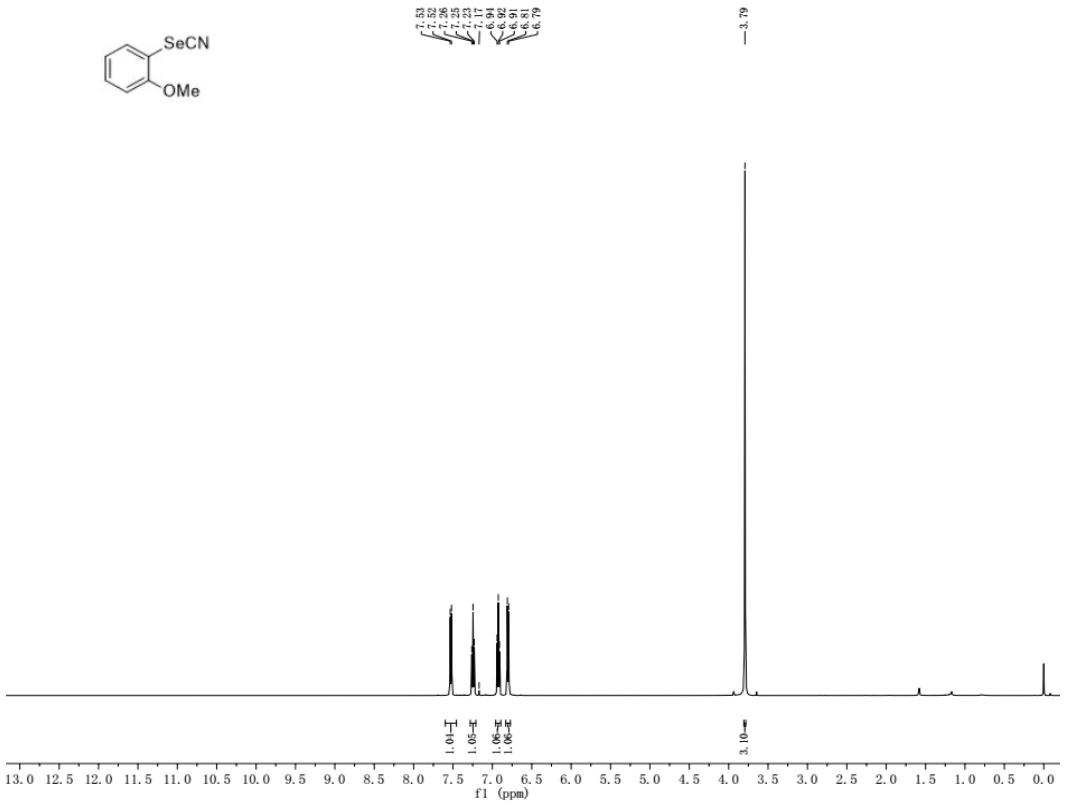

^{1}H NMR of compound **3q**

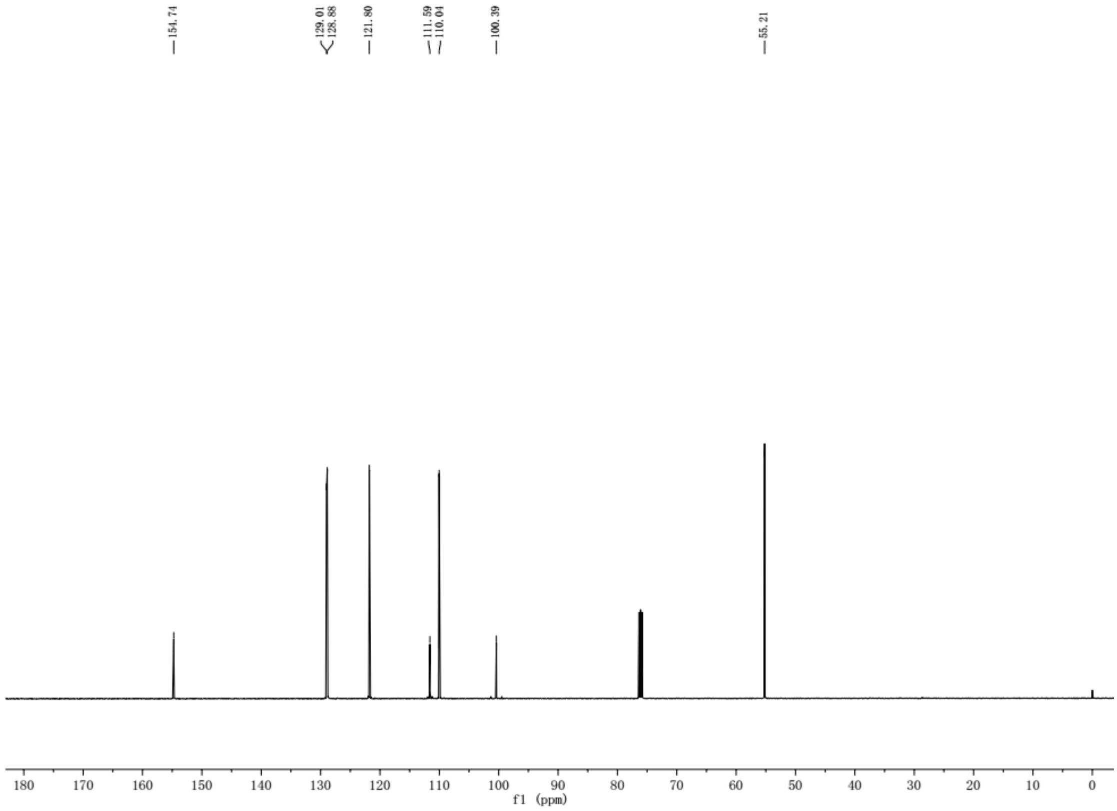

^{13}C NMR of compound **3q**

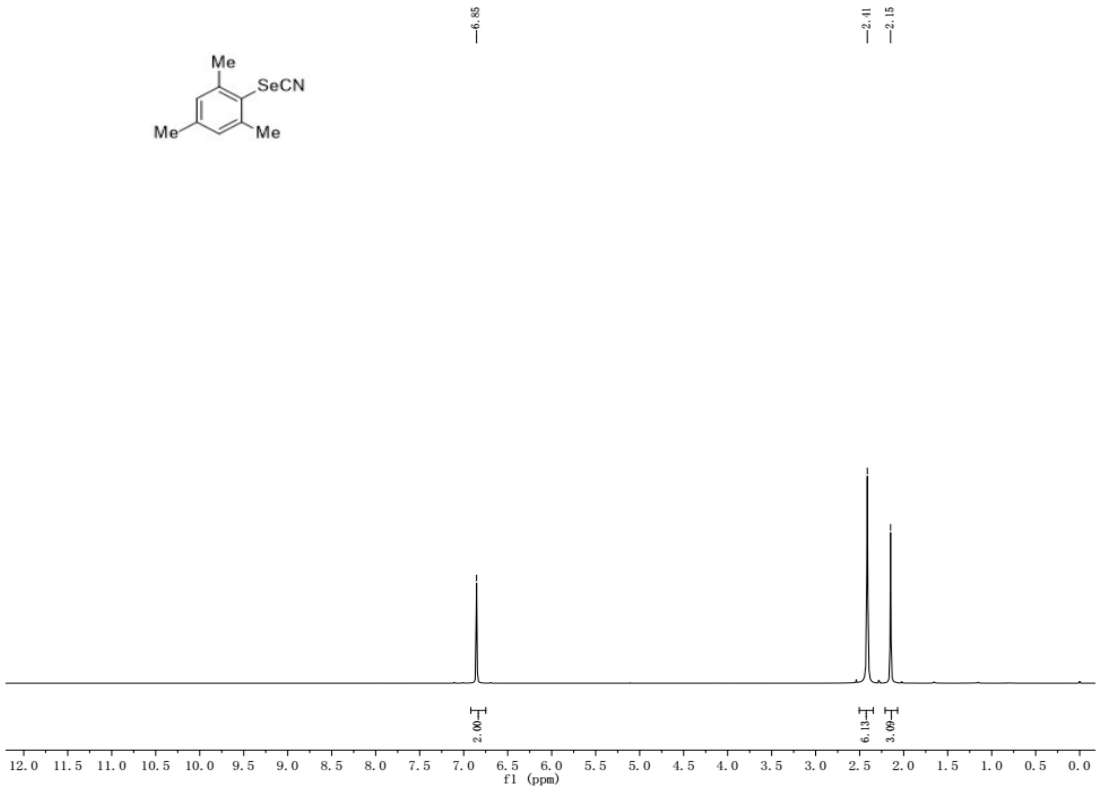

^{1}H NMR of compound **3r**

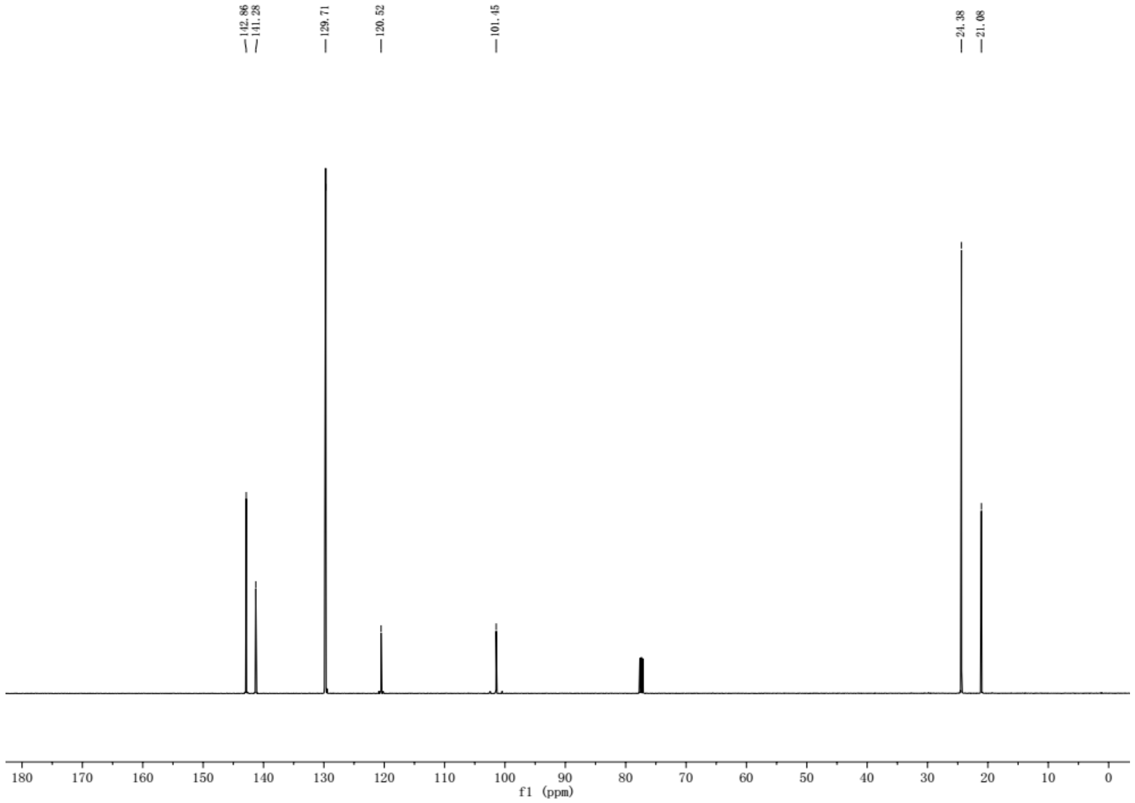

^{13}C NMR of compound **3r**

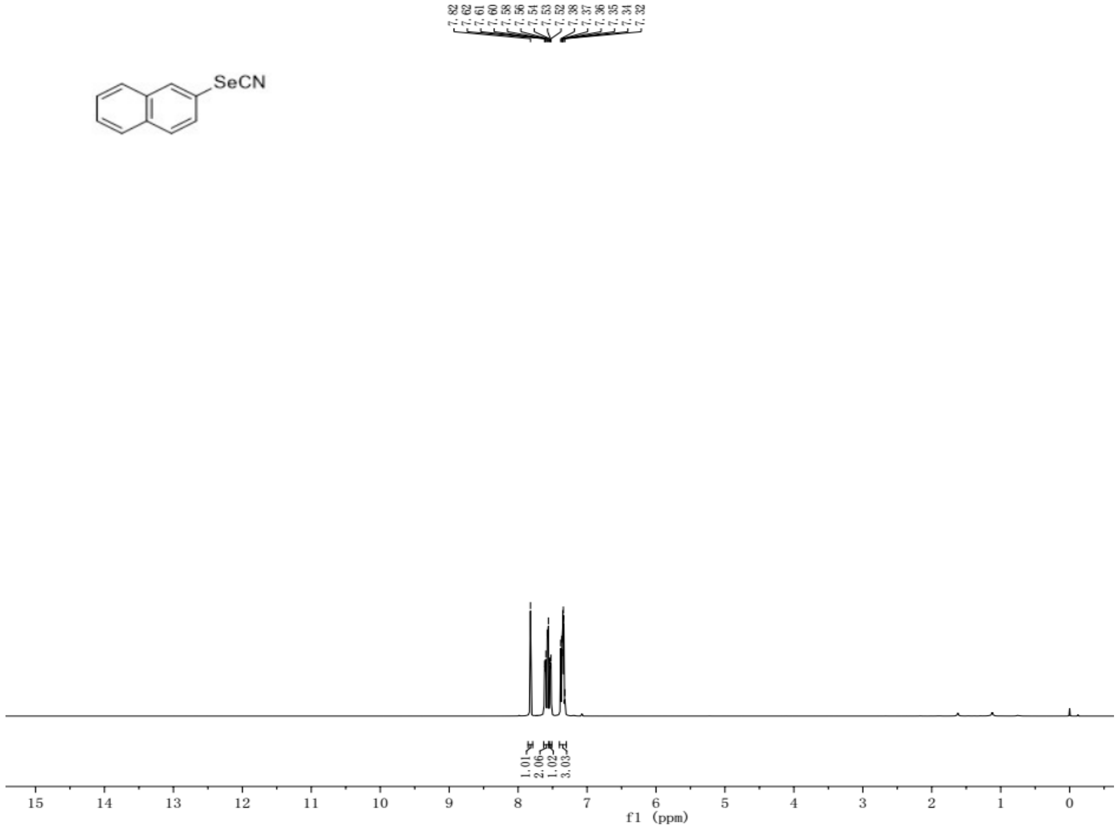

^{1}H NMR of compound **3s**

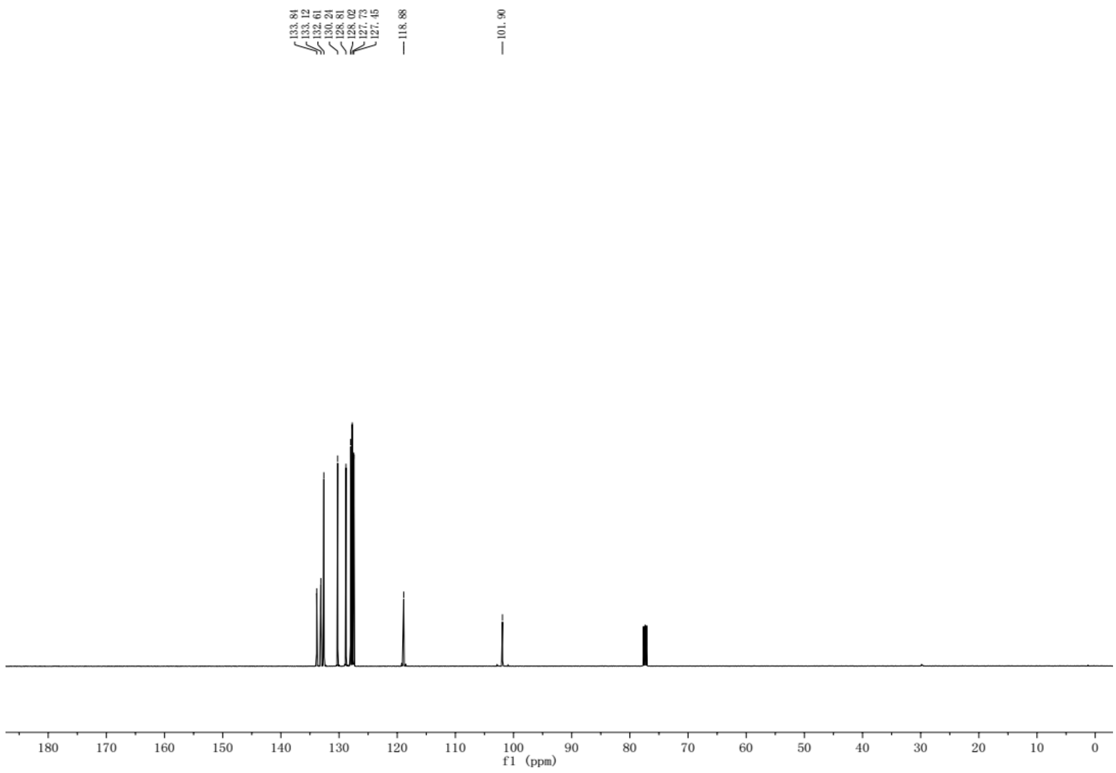

^{13}C NMR of compound **3s**

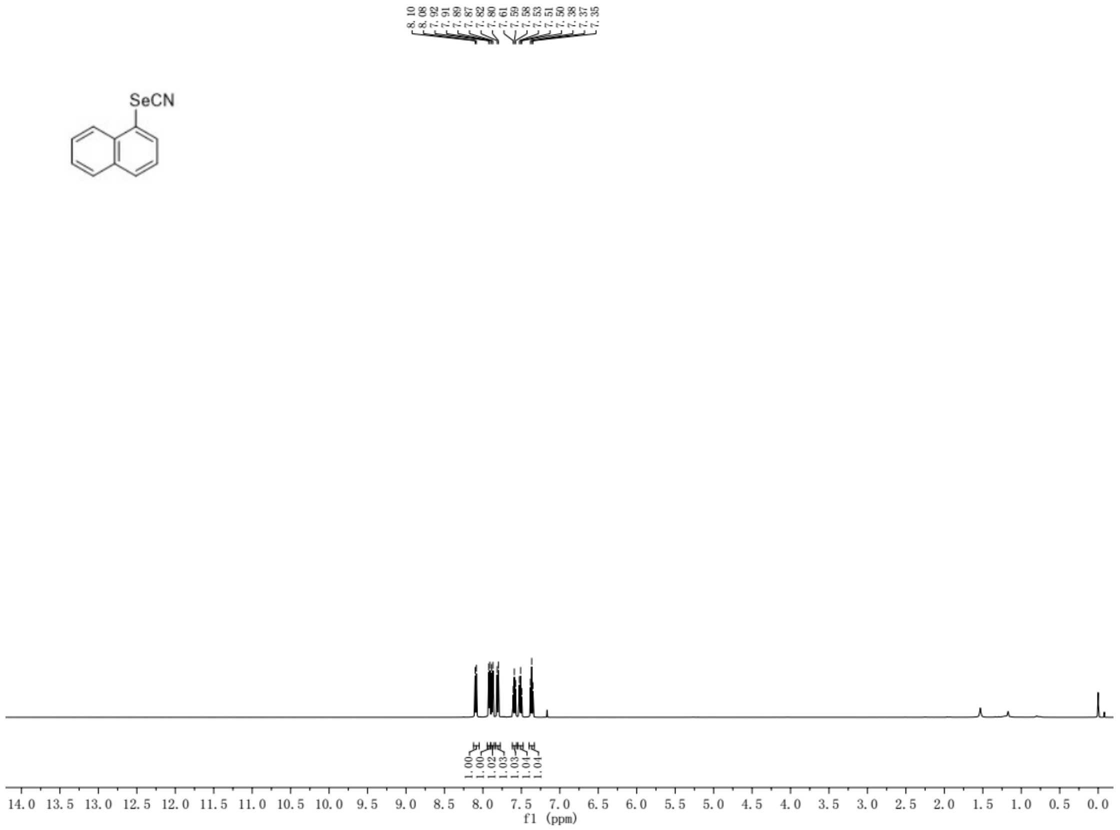

^{1}H NMR of compound **3t**

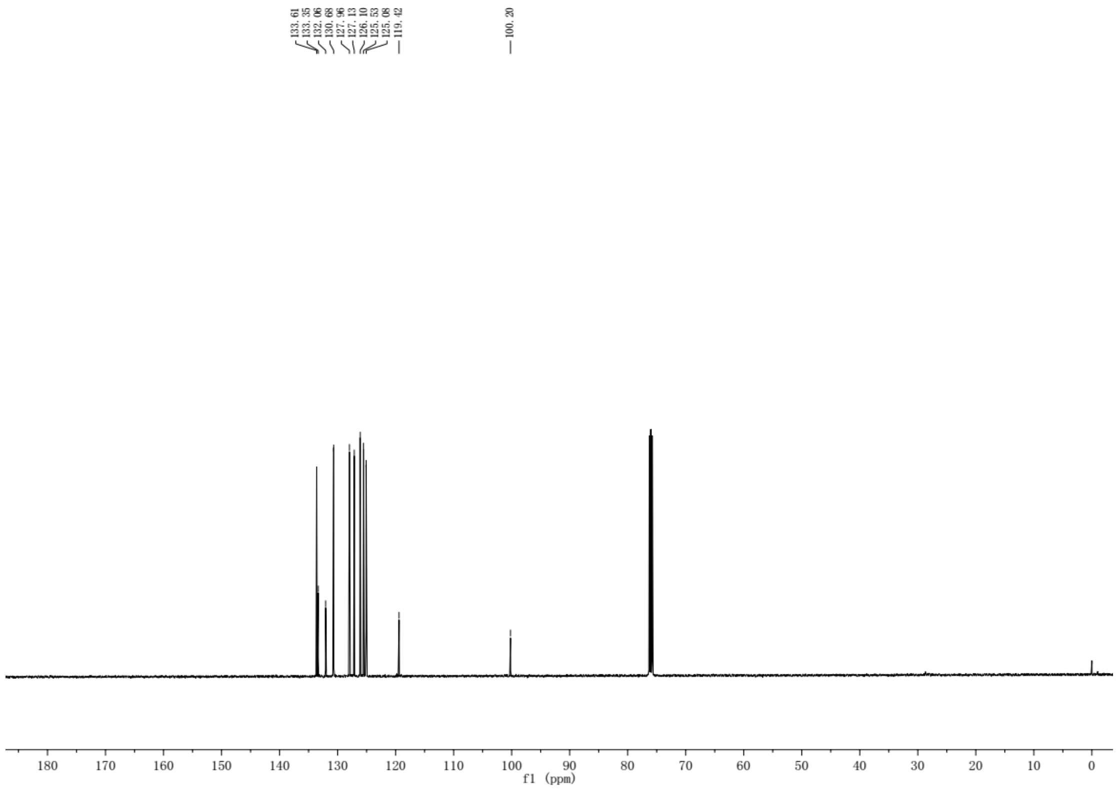

^{13}C NMR of compound **3t**

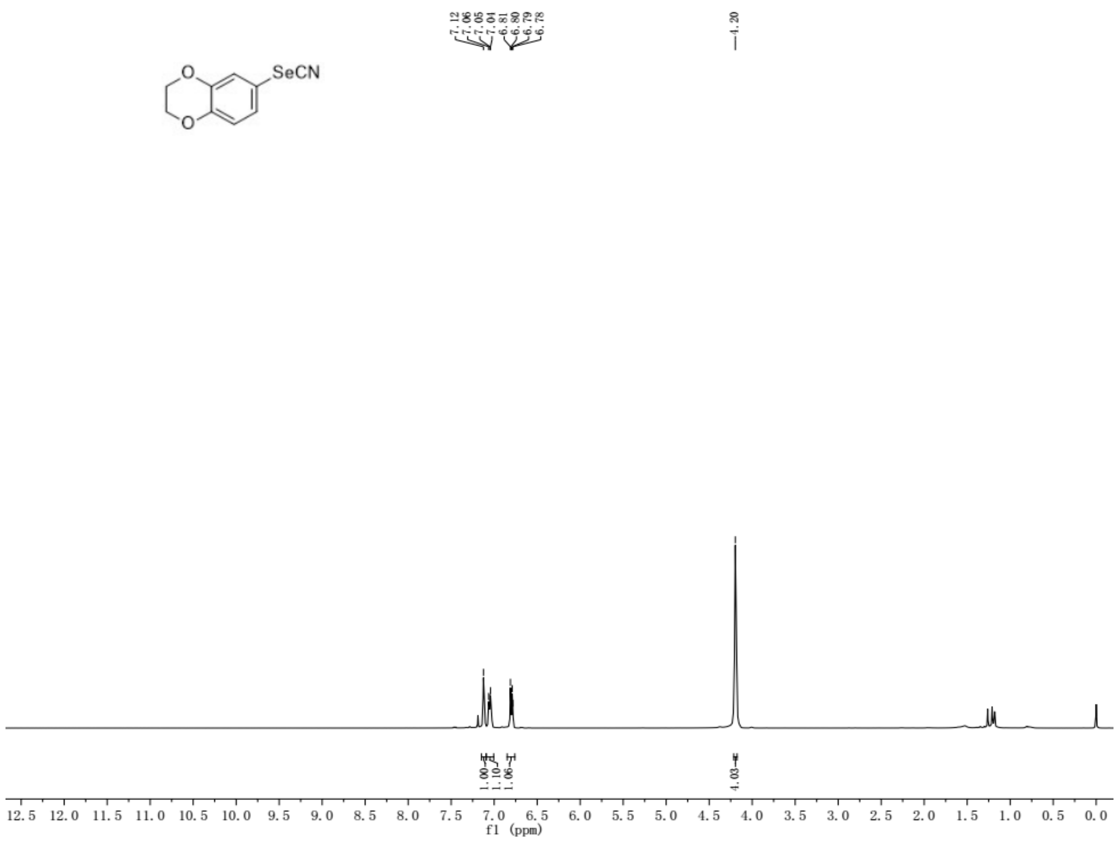

^{1}H NMR of compound **3u**

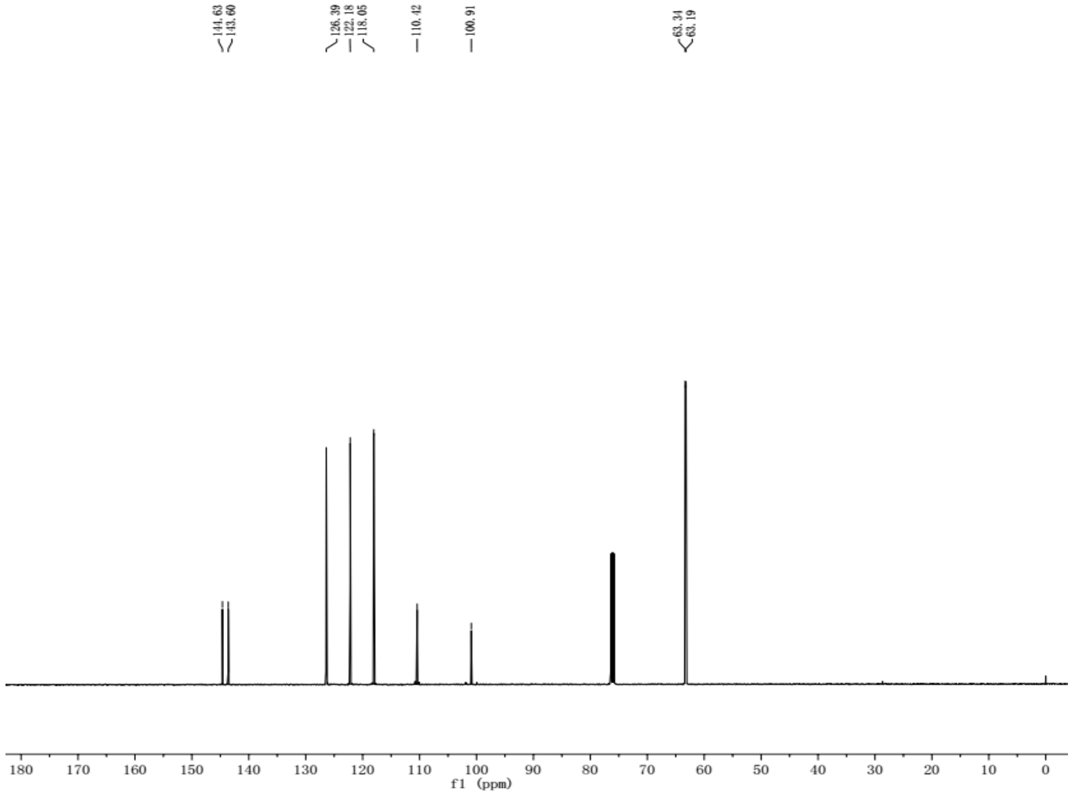

^{13}C NMR of compound **3u**

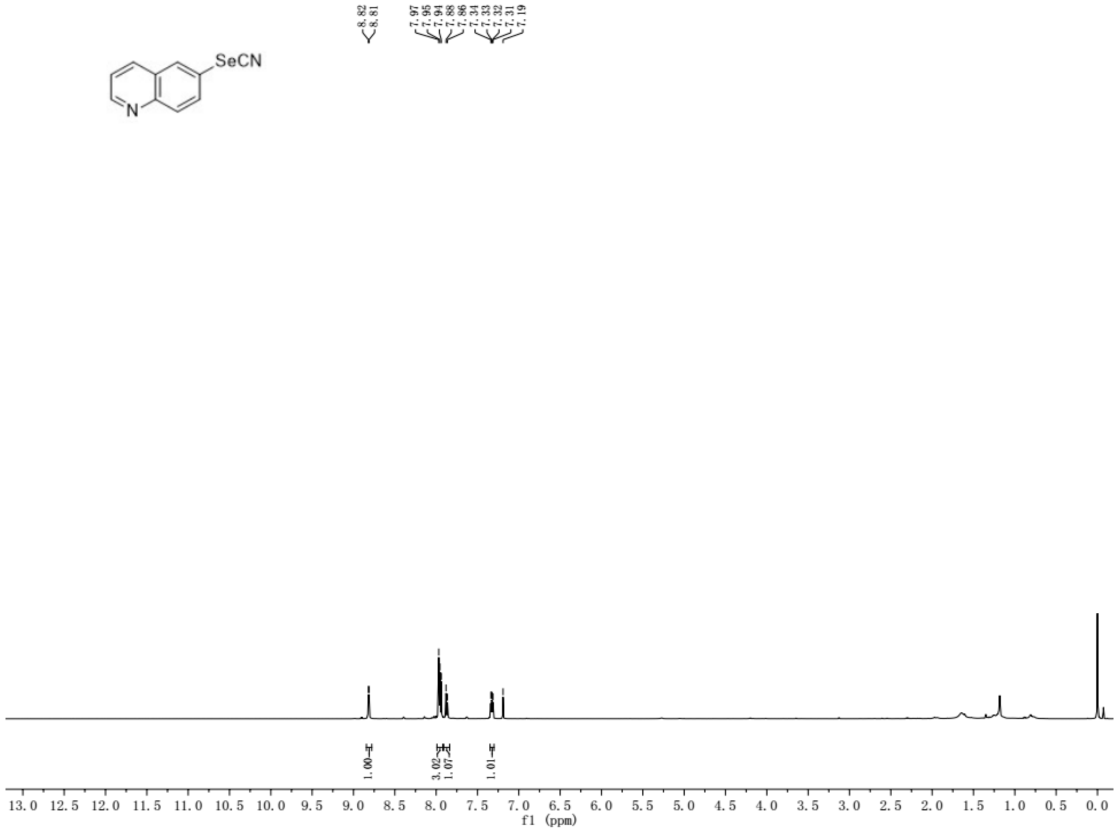

^{1}H NMR of compound **3v**

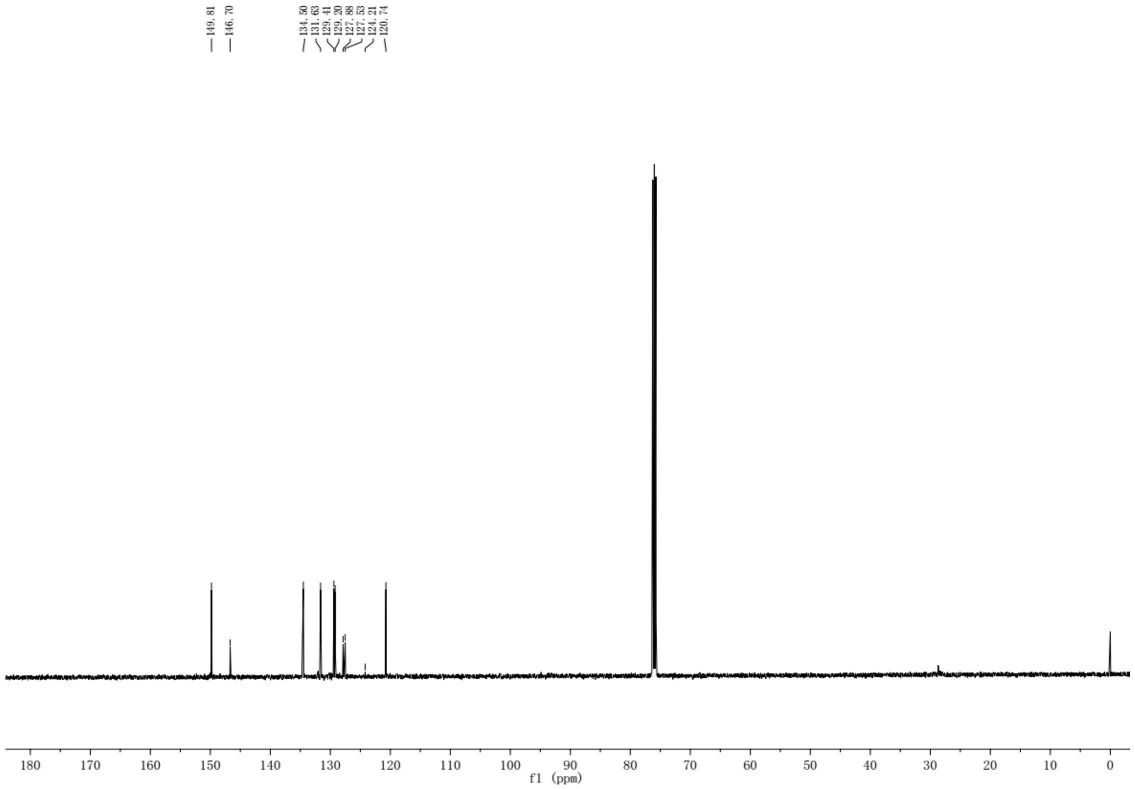

^{13}C NMR of compound **3v**

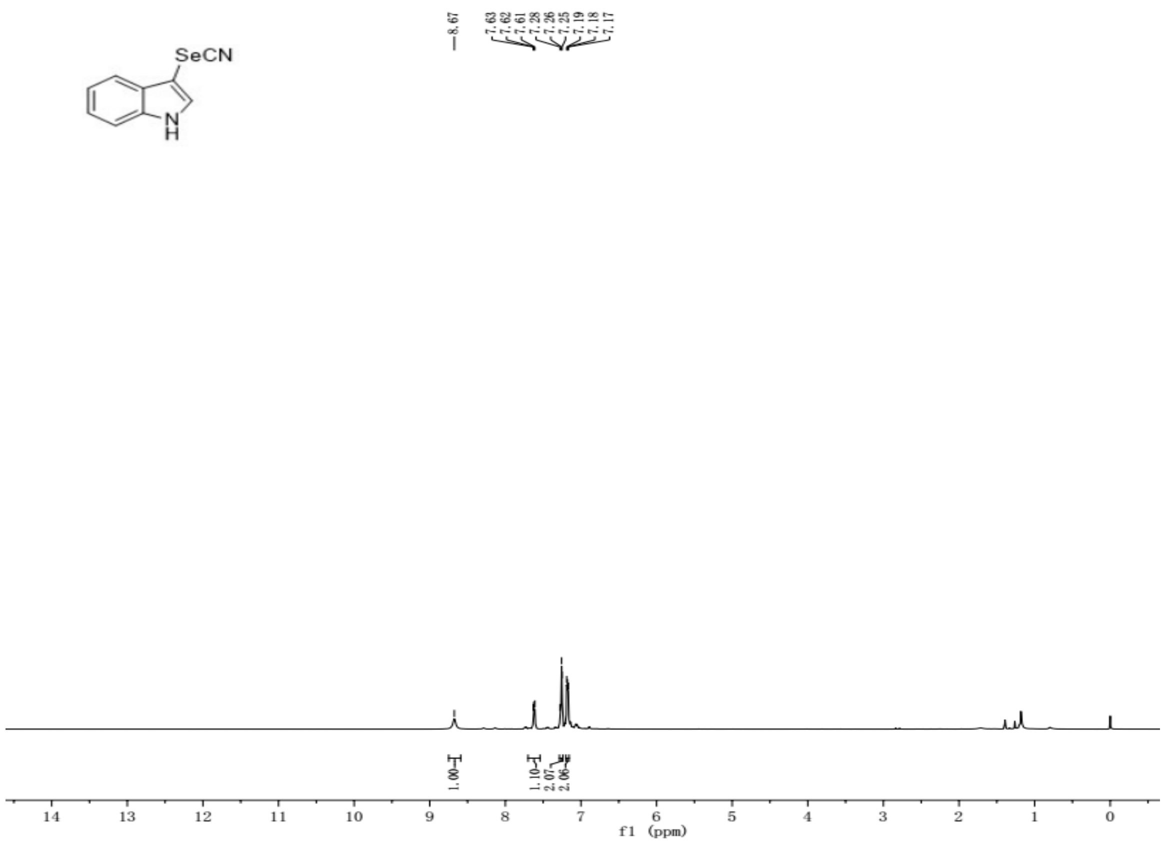

^{1}H NMR of compound **3w**

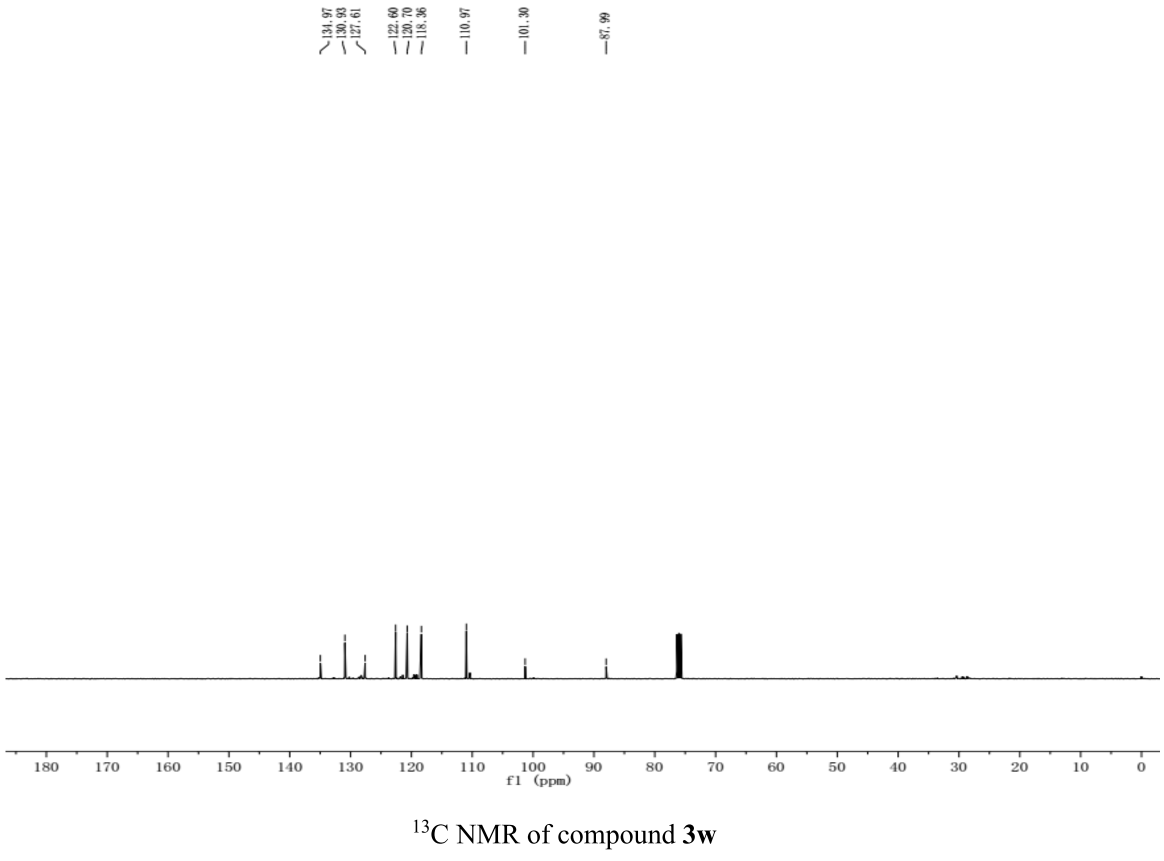

^{13}C NMR of compound **3w**

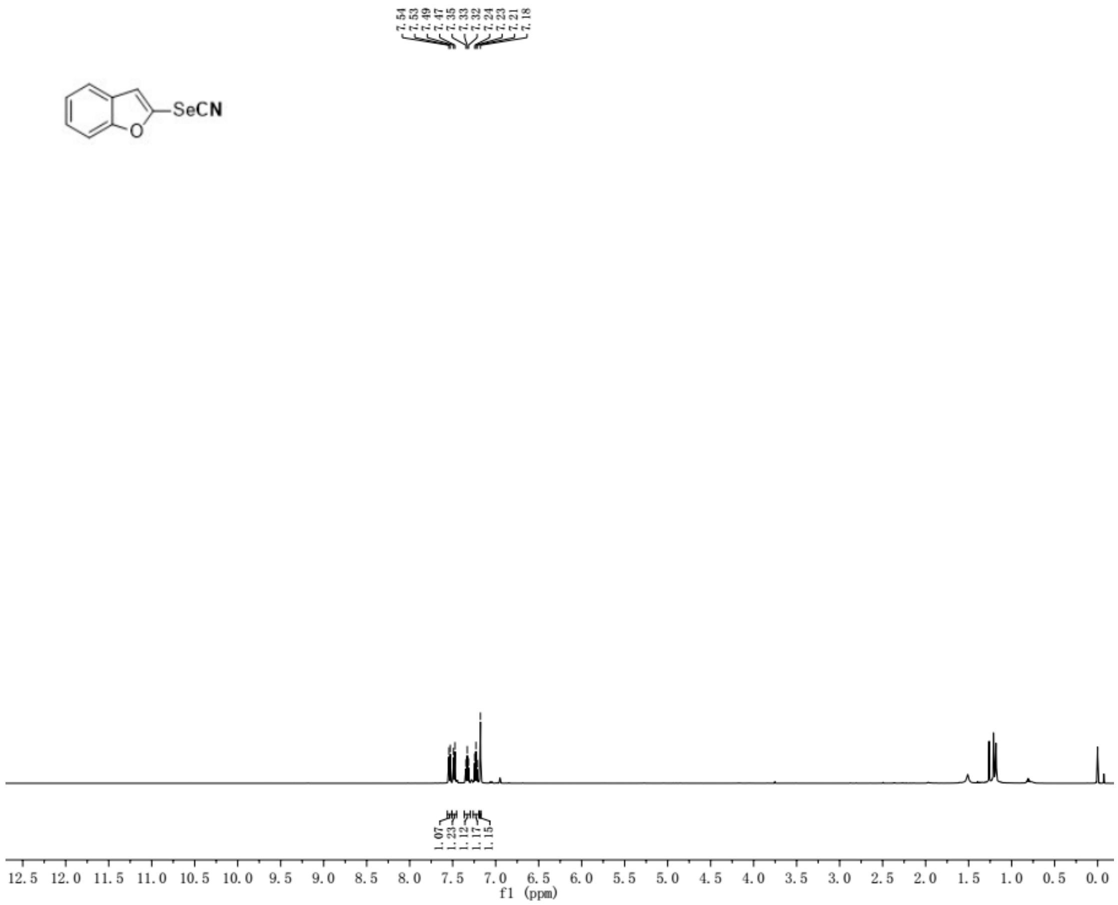

^{1}H NMR of compound **3x**

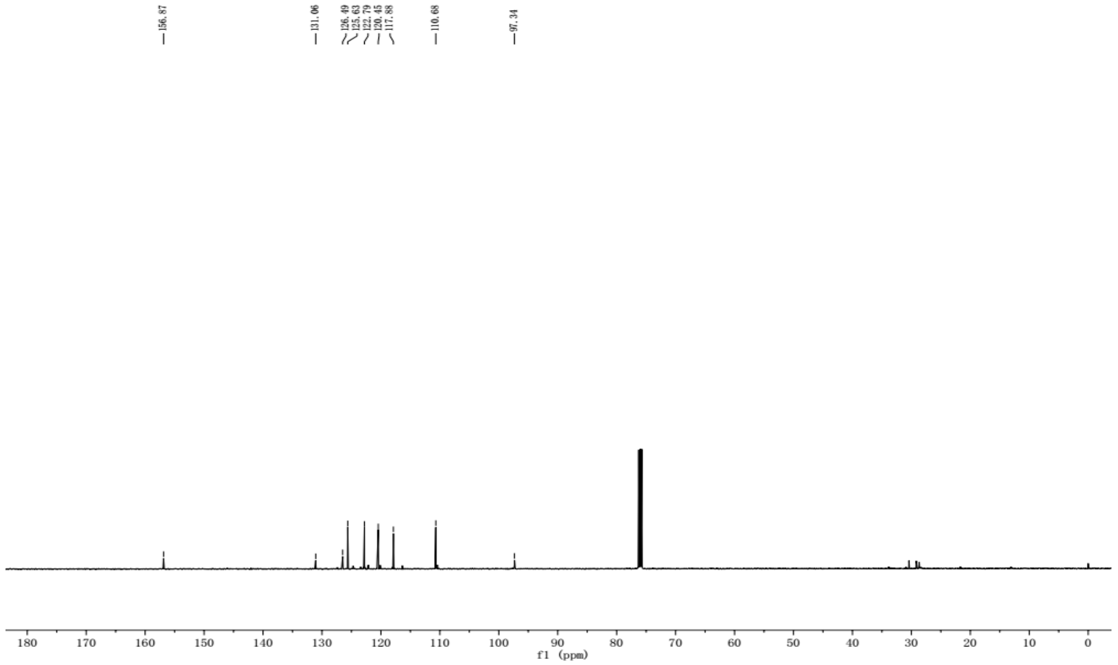

^{13}C NMR of compound **3x**
